# The endophytic symbiont *Epichloë festucae* establishes an epiphyllous net on the surface of *Lolium perenne* leaves by development of an expressorium, an appressorium‐like leaf exit structure

**DOI:** 10.1111/nph.13931

**Published:** 2016-03-17

**Authors:** Matthias Becker, Yvonne Becker, Kimberly Green, Barry Scott

**Affiliations:** ^1^ Institute of Fundamental SciencesMassey UniversityPalmerston North4442New Zealand; ^2^ IGZ – Leibniz Institute of Vegetable and Ornamental Crops14979GroßbeerenGermany; ^3^ Bioprotection Research CentreMassey UniversityPalmerston North4442New Zealand

**Keywords:** endophyte, epiphyte, expressorium, fungal symbiosis, NADPH oxidase

## Abstract

- *Epichloë festucae* forms a mutualistic symbiotic association with *Lolium perenne*. This biotrophic fungus systemically colonizes the intercellular spaces of aerial tissues to form an endophytic hyphal network. *E. festucae* also grows as an epiphyte, but the mechanism for leaf surface colonization is not known. Here we identify an appressorium‐like structure, which we call an expressorium that allows endophytic hyphae to penetrate the cuticle from the inside of the leaf to establish an epiphytic hyphal net on the surface of the leaf.
- We used a combination of scanning electron, transmission electron and confocal laser scanning microscopy to characterize this novel fungal structure and determine the composition of the hyphal cell wall using aniline blue and wheat germ agglutinin labelled with Alexafluor‐488.
- Expressoria differentiate immediately below the cuticle in the leaf blade and leaf sheath intercalary cell division zones where the hyphae grow by tip growth. Differentiation of this structure requires components of both the NoxA and NoxB NADPH oxidase complexes. Major remodelling of the hyphal cell wall occurs following exit from the leaf.
- These results establish that the symbiotic association of *E. festucae* with *L. perenne* involves an interconnected hyphal network of both endophytic and epiphytic hyphae.

## Abstract 2

See also the Commentary on this article by Genre & Lanfranco, **211**: 5–7.

## Introduction

*Epichloë festucae* (Ascomycota, Clavicipitaceae) forms mutualistic symbiotic associations with temperate grasses of the *Lolium* and *Festuca* genera (Leuchtmann *et al*., 1994). Natural hosts for *E. festucae* include *Festuca rubra* (fine fescue) and *Festuca longifolia* (hard fescue), but some strains also form stable associations with *Lolium perenne* (perennial ryegrass), an ideal host to study fungal endophyte–grass interactions (Christensen *et al*., 1997; Scott *et al*., 2012). *E. festucae* systemically colonizes the intercellular spaces of the aerial tissues of grasses, including leaf sheath and blade tissue (Tanaka *et al*., 2006; Scott *et al*., 2012) and the inflorescence tissues of reproductive tillers (May *et al*., 2008). Benefits to the fungal symbiont include access to nutrients and dissemination through the host seed, whereas the host benefits through increased tolerance to biotic and abiotic stresses (Schardl *et al*., 2009).

*Epichloë festucae* colonization of *L. perenne* and establishment of a mutualistic symbiotic interaction are tightly synchronized with development of the grass host. New leaf growth occurs at the base of the plant in the shoot apical meristem (SAM), which gives rise to leaf primordia and axillary buds. *E. festucae* grows between these undifferentiated plant cells by hyphal tip growth and branching, and consequently newly developing leaves are continuously colonized by hyphae (Christensen & Voissey, 2007). As leaf primordia of grasses develop, they differentiate into two tissues of limited cell division, the blade and sheath intercalary division zones (Christensen *et al*., 2008; Scott *et al*., 2012). Leaf intercellular spaces start to develop at junctions of mesophyll initials (Giannoutsou *et al*., 2013). This early established continuum of leaf intercellular spaces is colonized by profusely branching hyphae of *E. festucae* that become attached to plant cell walls.

In contrast to the highly branched hyphal network that forms within tissues of dividing plant cells, hyphae colonizing the intercellular cavities of expanding and differentiating tissue of sheath and blade are restricted in growth, as the number of hyphae do not increase further (Tan *et al*., 2001; Christensen *et al*., 2002). Hyphae appear to be tightly attached to the plant cells by an adhesive matrix, so that when plant cells elongate in the leaf expansion zone, hyphae become stretched and switch from tip growth to intercalary growth (Christensen *et al*., 2008; Voisey, 2010). Intercalary growth of hyphae stops when leaf expansion ceases but the fungus remains metabolically active and hyphal diameter increases (Tan *et al*., 2001). This pattern of growth explains why hyphae within a distinct developmental zone of the leaf have the same morphology and physiological state (Christensen *et al*., 2008).

Besides growing endophytically, *E. festucae* can also grow epiphytically on the surface of grass leaves following emergence of the hyphae from between epidermal cells in the lower part of the leaf expansion zone (Christensen *et al*., 1997; Tanaka *et al*., 2006; Christensen & Voissey, 2007; Eaton *et al*., 2010; Scott *et al*., 2012). In contrast to the dense mycelial layer associated with stromata formation and the associated ‘choke’ disease on reproductive tillers (Schardl, 2001), leaves with epiphytic hyphal growth remain symptomless (Christensen & Voissey, 2007). Importantly, these epiphytic hyphae remain connected to the endophytic hyphal network, and like the latter, stop growing when the leaf is fully elongated (Christensen & Voissey, 2007). Thus, *E. festucae* may be considered to be both an epiphyte and an endophyte. While the benefits of endophytes to plant health and growth are well documented, much less is known about the benefits of epiphytic microbes (Leveau & Lindow, 2001; Yang *et al*., 2001). The presence of extensive hyphal nets of *Epichloë typhina* on the surface of *Poa ampla* leaves has led to the attractive hypothesis that epiphyllous hyphae may increase the resistance of the host to fungal pathogens through ‘niche exclusion’ (Moy *et al*., 2000).

The synchronization of *E. festucae* hyphal growth with leaf growth implies there must be signalling between the two partners to maintain such a tightly regulated symbiotic state. From a series of forward and reverse genetic screens, we have shown that disruption of genes encoding the NADPH oxidase complex components NoxA, NoxR and RacA (Takemoto *et al*., 2006; Tanaka *et al*., 2006, 2008), the transcription factor ProA (Tanaka *et al*., 2013), the stress‐activated (SakA) and cell wall integrity (MkkA and MpkA) mitogen‐activated protein (MAP) kinase pathways (Eaton *et al*., 2010; Becker *et al*., 2015), leads to a severe host interaction phenotype, including severe stunting, hypertillering and premature senescence. Mutant hyphae in these associations were hyperbranched in the leaves and no longer aligned parallel to the leaf axis, vascular bundles were infected, and fungal biomass was significantly increased. This pattern of proliferative growth contrasts with the restrictive growth pattern observed for the wild‐type strain.

The objective of this study was to understand how *E. festucae* establishes an epiphyllous hyphal net on leaf blade and sheath tissue when in symbiotic association with *L. perenne*. While previous studies have shown that *E. festucae* develops epiphyllous hyphae (Christensen *et al*., 1997; Eaton *et al*., 2010; Scott *et al*., 2012), the mechanism for colonizing the leaf surface of the host is not known. Here, we describe a novel appresorium‐like structure for exiting leaves and show that NoxA, NoxB and NoxR are required for this developmental process. The results of this study suggest that growth and development of epiphyllous hyphae, like those of endophytic hyphae, are restricted so that a balanced plant–fungus interaction occurs to maintain a mutualistic symbiotic association.

## Materials and Methods

### Strains and growth conditions

Cultures of *Epichloё festucae* (Supporting Information Table S1), including wild‐type strain Fl1 (PN2278), Δ*noxA* (PN2327), Δ*noxB* (PN2469), Δ*noxAB* (PN2470) and Δ*noxR* (PN2497) mutants (Takemoto *et al*., 2006; Tanaka *et al*., 2006), were grown on 2.4% potato dextrose (PD) agar and 1.5% water agar under conditions previously described (Moon *et al*., 1999, 2000). For microscopy analyses of hyphal structures in culture, mycelium was taken from cultures grown for 10 d on PD agar plates. A description of all biological material is provided in Table S1.

### Plant growth and endophyte inoculation conditions

Endophyte‐free seedlings of perennial ryegrass (*Lolium perenne* cv Samson) were inoculated with *E. festucae* using the method of Latch & Christensen (1985). For examination of epiphyllous growth, seedlings were grown on Murashige and Skoog‐phytoagar (0.75% (w/v) phytoagar, 0.43% (w/v) Murashige and Skoog medium) under sterile conditions as well as under nonsterile conditions in root trainers with potting mix in an environmentally controlled growth room at 22°C with a photoperiod of 16 h of light (*c*. 100 μmol m^−2^ s^−1^). Host colonization characteristics of *E. festucae* wild‐type and mutant strains and *L. perenne* plant phenotype were similar under sterile and nonsterile conditions. Leaf samples were taken 8–12 wk after inoculation of ryegrass seedlings. Samples were taken from at least two tillers per plant and at least three plants per growth condition. Only turgescent leaves with a visible ligule were used (two to four per tiller). Adaxial (upper) and abaxial (lower) surfaces of the leaf blade, leaf blade expansion zone and sheath were examined.

### Light microscopy

For examination of fungal growth on leaves, tissues were stained with lactophenol Trypan Blue as previously described (Koch & Slusarenko, 1990) and viewed using a Zeiss Axiophot light microscope (Jena, Germany). Images were recorded using a Leica DCF320 digital camera.

### Confocal laser scanning microscopy

Growth and morphology of *E. festucae* hyphae *in planta* were determined by staining leaves with aniline blue diammonium salt (AB; Sigma‐Aldrich) in combination with wheat germ agglutinin‐conjugated Alexa Fluor^®^488‐(WGA‐AF488; Molecular Probes, Eugene, OR, USA) or Trypan Blue BDH Chemicals by VWR (Radnor, PA, USA) as follows. To stain with Trypan Blue, tillers of perennial ryegrass infected with *E. festucae* were soaked in 95% (v/v) methanol for 24 h, with one change of methanol after 12 h. Leaves were boiled for 5 min in 1 ml lactophenol Trypan Blue solution (10 ml lactic acid, 10 ml glycerol, 10 g phenol, 10 mg Trypan Blue, 10 ml water), and samples were transferred into fixing solution (50 g chloralhydrate in 50 ml water) for 30 min (Koch & Slusarenko, 1990). To stain with aniline blue/WGA‐AF488, tillers of perennial ryegrass infected with *E. festucae* were soaked in 95% (v/v) ethanol overnight at 4°C, and then treated with 10% potassium hydroxide for 3 h. The tissue was washed three times with phosphate‐buffered saline (PBS, pH 7.4) and incubated in staining solution (0.02% aniline blue, 10 ng ml^−1^ WGA‐AF488, and 0.02% Tween 20 in PBS (pH 7.4)) for 5 min, followed by 30 min vacuum infiltration). Fluorescence was recorded with a Leica SP5 DM6000B confocal laser scanning microscope (CLSM) (×10, numerical aperture (NA) 0.4; ×20, NA 0.7; ×40 oil immersion objective, NA 1.3, ×63 oil immersion objective, NA 1.4) (Leica Microsystems). Hyphae stained with Trypan Blue were excited with a krypton‐argon laser at 561 and 633 nm, respectively. The AB/WGA‐AF488 dyes were excited simultanously with the 488 nm argon ion laser and 561 nm diode‐pumped solid‐state (DPSS) laser. Three photomultiplier tubes (PMTs) were used to capture the emission fluorescence from the dyes as well as plant autofluorescence and pseudocolours used to represent specific emission fluorescence. Blue pseudocolour (PMT1, 498–551 nm) was assigned to emission fluorescence from WGA‐AF488 excited with the 488 nm argon ion laser and two different pseudocolours were assigned to PMTs (PMT2, 571–633 nm green, and PMT3, 661–800 nm red) for emission fluorescence from aniline blue and plant autofluorescence resulting from excitation with the 561 nm DPSS laser. This enabled us to: distinguish hyphal cell wall from protoplast (pseudocoloured in red and orange); capture changes in fungal cell wall composition (shift in red to blue pseudocolour as a result of WGA‐AF488 chitin labelling); and visualize the cuticle (pseudocoloured in yellow‐green). By collecting a combination of light emitted from dyes and plant autofluorescence, we managed to get a strong and specific signal from the plant cuticle. This overcame the need to use a specific dye to detect lipophilic components, which might have interfered with WGA‐AF488 or aniline blue imaging. Image analysis was done with LAS AF and ImageJ. Composite images of *z*‐series of images are shown as maximum projections.

### Scanning electron microscopy

Leaf samples examined by scanning electron microscopy (SEM) were incubated in fixative (3% glutaraldehyde and 2% formaldehyde in 0.1 M phosphate buffer, pH 7.2) for a minimum of 24 h at room temperature. Samples were washed three times with 0.1 M phosphate buffer (pH 7.2) and dehydrated by passing through a graded ethanol series. Culture samples were not fixed but just dehydrated through a graded ethanol series. Plant and culture samples were then critical point‐dried using liquid CO_2_, mounted onto aluminium specimen support stubs, sputter‐coated with gold, and observed using an field electron and ion (FEI Quanta 200; FEI Co., Hillsboro, OR, USA) scanning electron microscope.

### Transmission electron microscopy

For transmission electron microscopy (TEM), pseudostem sections were fixed in 3% glutaraldehyde and 2% formaldehyde in 0.1 M phosphate buffer, pH 7.2, for 1 h as described by Spiers & Hopcroft (1993). A Philips CM10 (Amsterdam, the Netherlands) and a FEI Tecnai G2 Spirit BioTWIN TEM (FEI Co.) were used to examine the fixed samples.

### Generation of new *noxB* mutants

Phusion High‐Fidelity DNA polymerase (New England Biolabs, Ipswich, MA, USA) was used to amplify a 4.7 kb replacement fragment with primers (YB168–YB169), comprising 820 bp 5′ of *noxB,* the *nptII* resistance cassette, and 1059 bp 3′ of *noxB* using the *noxB* replacement construct pPN78 (Tanaka *et al*., 2006) as template.

*Epichloë festucae* protoplasts were prepared as previously described (Young *et al*., 2005). Protoplasts were transformed with 3–5 μg of linear restriction enzyme digested DNA using the method previously described (Itoh *et al*., 1994). Transformants were selected on regeneration medium (RM; PD with 0.8 M sucrose) containing geneticin (200 μg ml^−1^) and nuclear purified by three rounds of subculturing on PD medium containing the same antibiotic selection.

Three independent ‘clean’ Δ*noxB* mutants were identified by PCR and Southern blot analysis, including PN3062 (ΔnoxB#1), PN3063 (ΔnoxB#14) and PN3064 (ΔnoxB#15). For the PCR analysis genomic DNA was screened for absence of internal *noxB* fragment using primer set YB167–YB151 and presence of 5′ and 3′ flanks with primer sets YB170–YB165 and YB166–YB171, respectively (Table S2).

## Results

### *Epichloë festucae* forms an expressorium, an appressorium‐like leaf exit structure

**Figure 1:**
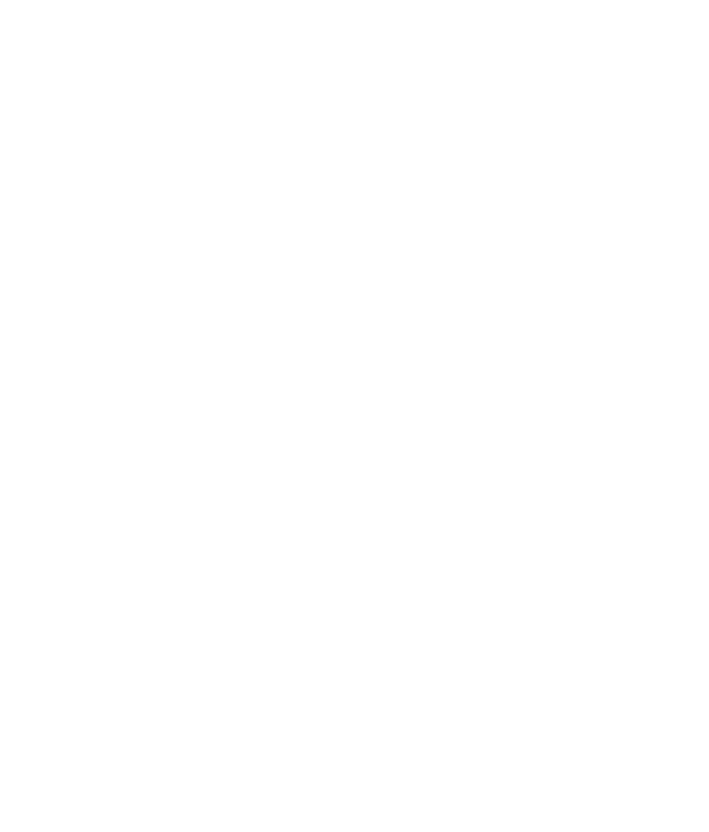
Plant interaction phenotype for *Lolium perenne* infected with *Epichloë festucae* wild‐type (WT) and NADPH oxidase complex mutants. (a) Whole‐plant phenotype of *E. festucae* wild‐type and ∆*noxA*, ∆*noxB*, ∆*noxAB* and ∆*noxR* mutants at 8 wk post inoculation. (b, c) Epiphyllous growth (fluorescence captured with blue pseudocolour) of WT and ∆*noxA E. festucae* on the blade extension zone of *L. perenne*, showing the enhanced growth of epiphyllous hyphae in the latter. (d–g) Leaf sheath and blade cellular phenotypes of *L. perenne* infected with *E. festucae* WT (d, e) and Δ*noxA* (f, g), showing the enhanced proliferation of hyphae in the latter. Fluorescence of endophytic hyphae was captured with orange pseudocolour (from aniline blue), apart from septa captured with blue pseudocolour (from WGA‐AF488). Confocal depth series images of leaf tissue, showing hyphae stained with aniline blue/WGA‐AF488 (b–g). Images were generated by maximum intensity projection of confocal z‐stacks. Bars: (b, c) 100 μm; (d–g) 25 μm.

**Figure 2:**
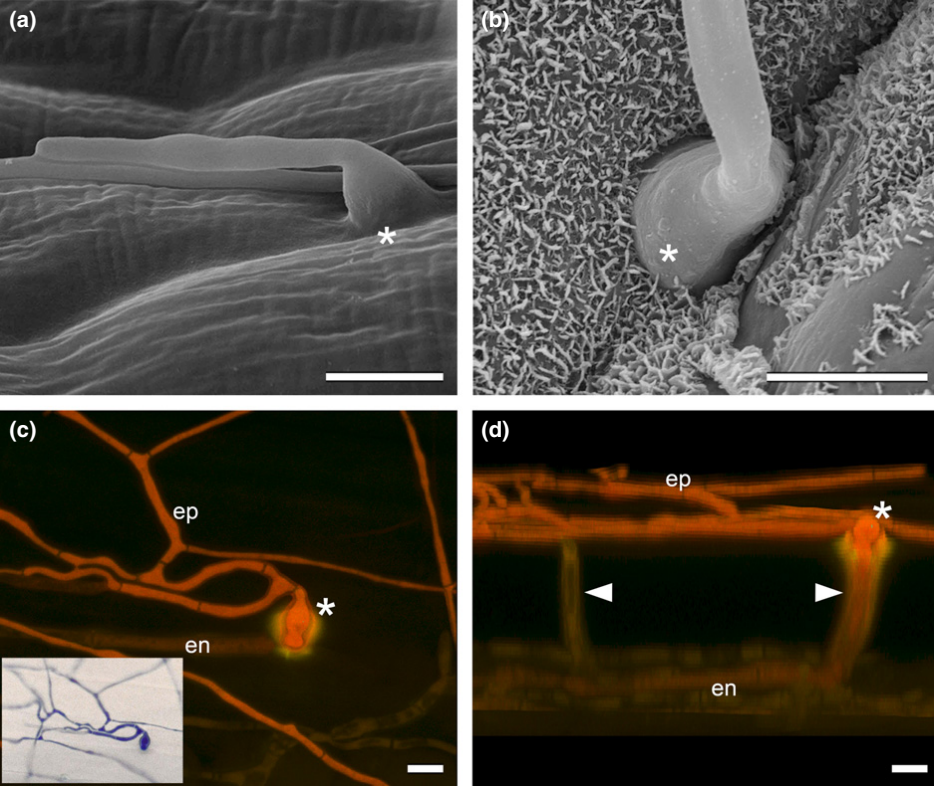
Expressoria of wild‐type *Epichloë festucae* in symbiosis with *Lolium perenne*. (a, b) Formation of expressoria (asterisks) on the adaxial leaf blade surface. (c) Image from above emerging hypha (asterisk) and epiphyllous hyphal net (ep) and light micrograph (window) showing a lower magnification of the same section. (d) Image from the side of (c), showing endophytic hyphae (en) and growth of a hypha between the epidermal cells (white arrowhead) and hyphal emergence (asterisk). Scanning electron microscopy (a, b) and maximum intensity projection *z*‐stack confocal laser scanning microscopy image (c, d) of Trypan Blue‐stained sample. Bars, 5 μm.

**Figure 3:**
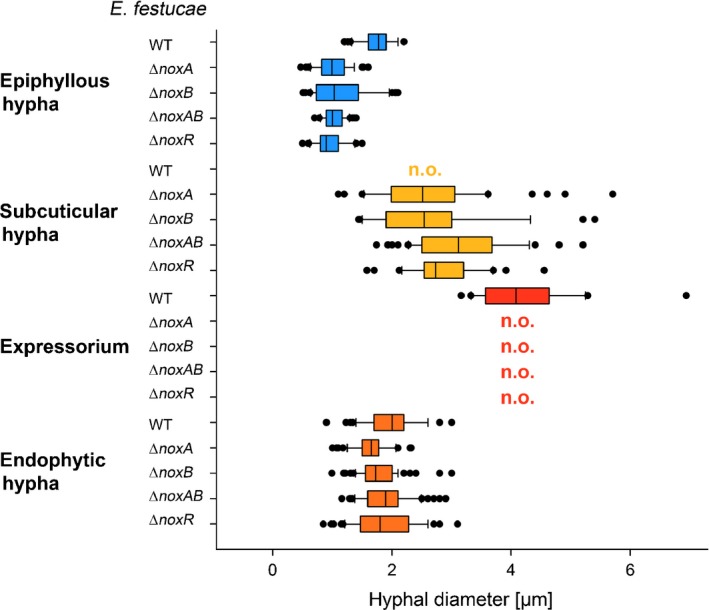
Diameters of *Epichloë festucae* wild‐type (WT) and *nox* mutant hyphae (in μm) based on confocal laser scanning microscopy (CLSM). Box plots showing average diameters and 95% confidence intervals of expressoria, subcuticular, endophytic, and epiphyllous hyphae (*n *=* *20–96), where the left and right edges of the boxes indicate the 25% and 75% quartiles, respectively, and the thick middle line indicates the median (50%). The whiskers correspond to the lowest and highest data points within the 1.5 interquartile range of the lower and upper quartiles, respectively. Outliers are indicated by closed circles. Expressoria (observed exclusively in WT) have larger hyphal diameters than other WT or *nox* mutant hypha. Subcuticular hyphae (regularly observed in *nox* mutants) are thicker and epiphyllous hyphae thinner than endophytic hyphae in *nox* mutants. WT endophytic and epiphyllous hyphae have similar diameters. Epiphyllous WT hyphae are significantly thicker than epiphyllous mutant hyphae (*P *<* *0.001 for all pairwise comparisons of epiphyllous wild‐type and *nox* mutant hyphae) as determined by one‐way ANOVA with Dunnett's test. n.o., not observed in CLSM.

**Figure 4:**
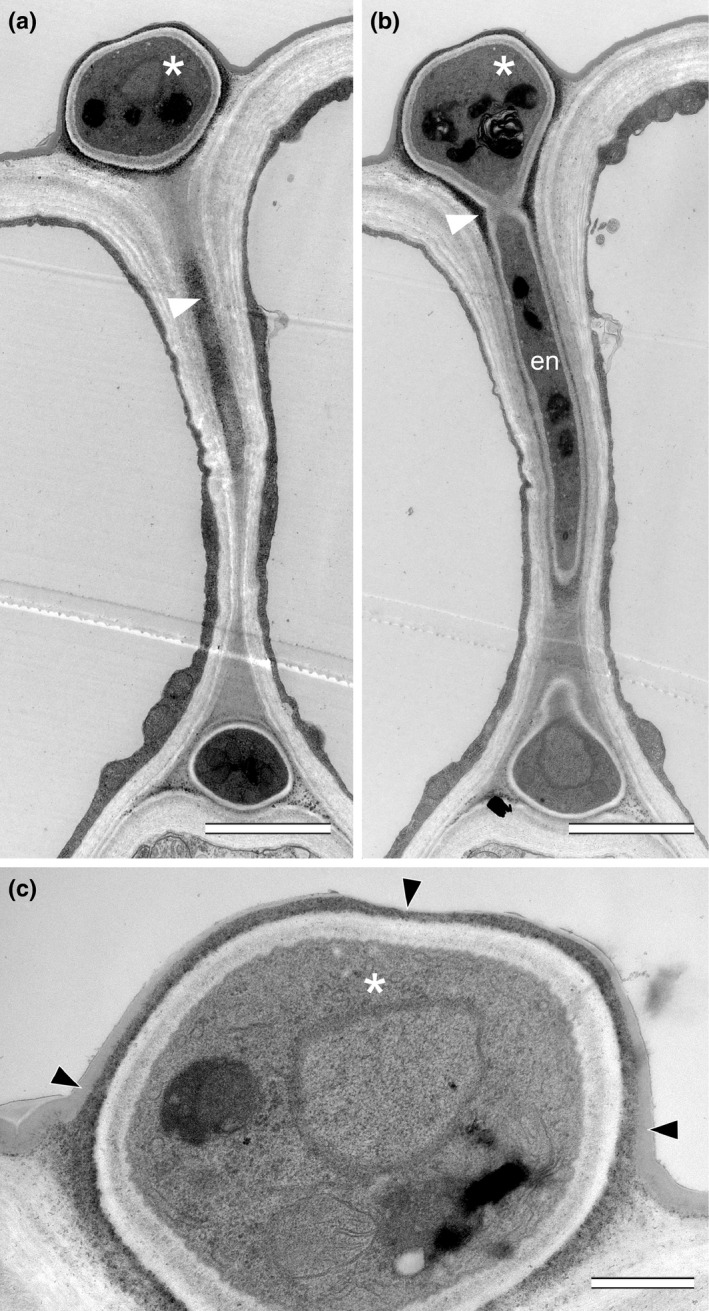
Transmission electron micrographs of wild‐type (WT) *Epichloë festucae* expressorium in symbiosis with *Lolium perenne*. (a, b) Serial slices of 100 nm cross‐sections of an endophytic fungal hypha (en) growing between two epidermal cells (white arrowheads) and formation of an expressorium (asterisk) below the leaf cuticle. The white arrowhead points to an electron‐dense layer surrounding WT hyphae, which is particularly thick at a constriction formed immediately below the hyphal swelling. (c) Higher magnification of expressorium shown in (a) and (b). The black arrowheads point to the cuticle, which is thinner on top of the expressorium. Bars: (a, b) 2 μm; (c) 0.5 μm.

**Figure 5:**
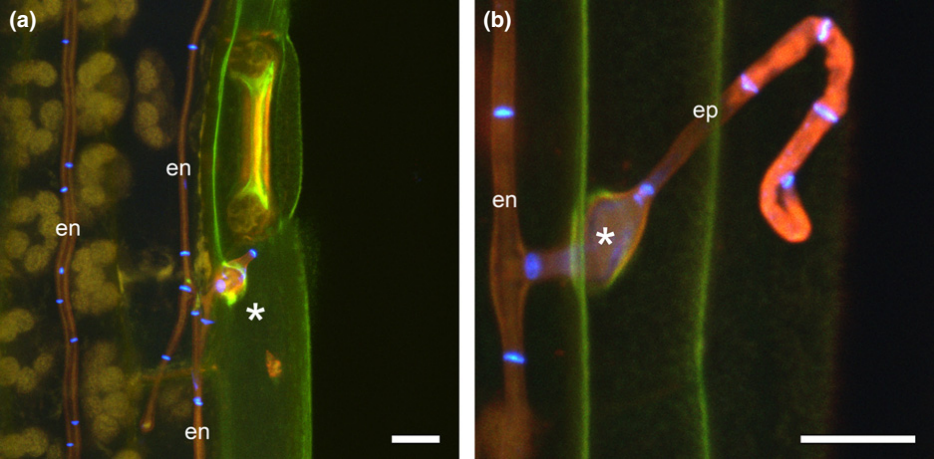
Wild‐type (WT) *Epichloë festucae* expressoria in symbiosis with *Lolium perenne*. (a) Endophytic hypha (en) and expressorium (asterisk) emergence close to a leaf blade stoma. Autofluorescence of chloroplasts and cuticle was captured as yellow and green pseudocolours. (b) Endophytic hypha emerging between epidermal cells to form a swollen expressorium (asterisk) and epiphyllous hypha (ep). Fluorescence from septa stained with WGA‐AF488 were captured in blue pseudocolour. Maximum intensity projection *z*‐stacks of confocal laser scanning microscopy images of aniline blue/WGA‐AF488 costained leaf blade sample. Bars, 10 μm.

**Figure 6:**
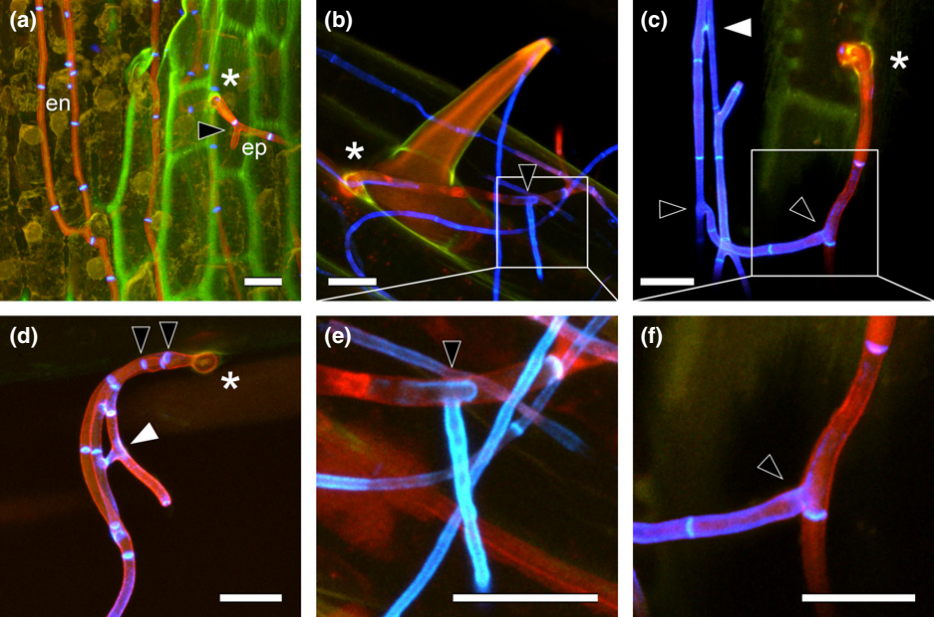
Cell wall alterations of wild‐type (WT) *Epichloë festucae* in symbiosis with *Lolium perenne* after hyphal penetration of plant cuticle and branching of epiphyllous hyphae. (a) Endophytic hyphae (en) exiting the plant by means of an expressorium (asterisk) and epiphyllous hypha (ep) branching soon after emergence on the surface of the leaf (black arrowhead). (b) Hypha emerging from base of a trichome (asterisk) with change in cell wall composition of epiphyllous hyphae revealed by WGA‐AF488 fluorescence captured in blue pseudocolour. (c, d) Hyphae emerging from between epidermal cells (asterisk), branching (black arrowheads) and fusing soon after emergence (white arrowhead). (e, f) Higher magnifications of (b) and (c) showing changes in cell wall composition, detected with WGA‐AF488 (shown in blue pseudocolour). Maximum intensity projection *z*‐stacks of confocal laser scanning microscopy images of aniline blue/WGA‐AF488 costained leaf blade sample. Bars, 10 μm.

Although the *E. festucae* symbiosis with *L. perenne* is established by endophytic hyphae (Fig. 1), hyphae within the leaf were frequently observed to breach the plant cuticle to emerge and grow on the outer surface of leaf sheath and blade (Figs 1, 2). Combined observations from light, CLSM, SEM and TEM suggest that hyphae emerge from the leaves by formation of an internal appressorium‐like structure that allows local penetration of the cuticle. Convention would suggest this structure be called an ‘intrinsecus appressorium’, given that it exerts pressure from the inside, but we propose to use the working term ‘expressorium’ (plural expressoria) to distinguish it from the external penetration structure (‘extrinsecus appressorium’ or appressorium) used by plant pathogens to enter plants. Expressoria form in regions of aerial tissue where hyphae are growing by tip growth, such as meristematic zones (Fig. S1). This form of growth is necessary for the hyphae to grow between the epidermal cells immediately below the cuticle. Contact with the inside of the cuticle appears to trigger differentiation of these structures. Expressoria were particularly abundant on the adaxial side of the leaf expansion zone (Fig. 1b), where plant cells divide to form the leaf blade. Once hyphae move into the leaf expansion zone, they grow by intercalary rather than tip growth and so can no longer grow between epidermal cells (and thus cannot encounter the cuticle) to form expressoria. On average, five to 10 emerging hyphae were observed in the leaf expansion zone per leaf examined. Swollen hyphae ranging from 3 to 7 μm in diameter were observed wherever a hypha exited the leaf blade (Fig. 3). However, the degree of swelling was variable, which may be a consequence of the strength of the cuticle. Hyphae never grow through stomata but appear to emerge exclusively from depressions between epidermal plant cells (Fig. 2), but no degradation of the anticlinal cell walls was observed (Fig. 4), suggesting that hypha exit by a remodelling of the epidermal middle lamella. CLSM images of Trypan Blue‐stained samples (Fig. 2c,d; Movie S1) and TEM of leaf cross‐sections (Fig. 4) revealed that the swollen hyphal compartment of the expressorium develops just below the cuticle after the hypha has passed between the cells of the epidermis. The increased turgor of this swollen compartment presumably provides the necessary pressure for the hypha to penetrate the cuticle. At the point of hyphal emergence there was a thinning of the cuticle, presumably as a result of stretching or hydrolysis (Fig. 4c) and polarization of the expressorium (Movie S2). As expressoria were relatively difficult to find in TEM samples of leaf cross‐sections, we used CLSM of aniline blue/WGA‐AF488‐stained samples to analyse in more detail the structure of expressoria (Fig. 5; Movies S3, S4) and their relationship to endophytic and epiphyllous hyphae (Fig. 6). Using these fluorophores and the settings specified in the Materials and Methods section, we were able to visualize both plant and fungal cells, and sub‐/extracellular structures of both, including chloroplasts (yellow) and cuticle (green) of the plant, and cell wall (orange/red) and septa (blue) of the endophyte. Cell walls of endophytic hyphae were exclusively stained by aniline blue, and only septa bound WGA‐AF488. By contrast, both cell walls and septa of epiphyllous hyphae bound WGA‐AF488. This change in distribution of WGA‐AF488 binding to epiphyllous hyphae suggests there is a major remodelling of the fungal cell wall upon exiting the leaf. However, this change in fluorescence distribution does not occur immediately after hyphae penetrate the cuticle (Fig. 5; Movies S3, S4), but after the expressorium has repolarized and formed two to four epiphyllous hyphal compartments. Cell wall remodelling starts at septa and expands gradually from the cytosol to the outer cell wall (Fig. 6).

While emerging hyphae lose contact with the leaf surface, they do appear to undergo a tropic response, resulting in recontact with the leaf surface and ongoing growth across the surface of the leaf (Fig. 2a). While endophytic hyphae of *E. festucae* were seldom branched, epiphyllous hyphae by contrast were frequently branched. Branches were observed in the first compartment that emerges from the expressorium (Figs 2c, 6; Movie S1) and in many of the subsequent compartments. Tip‐to‐side cell–cell fusions were also frequently observed among the epiphyllous hyphae (Fig. 6c,d).

### Phenotype of *E. festucae* growing on the surface of leaf blade and sheath compared with axenic culture

**Figure 7:**
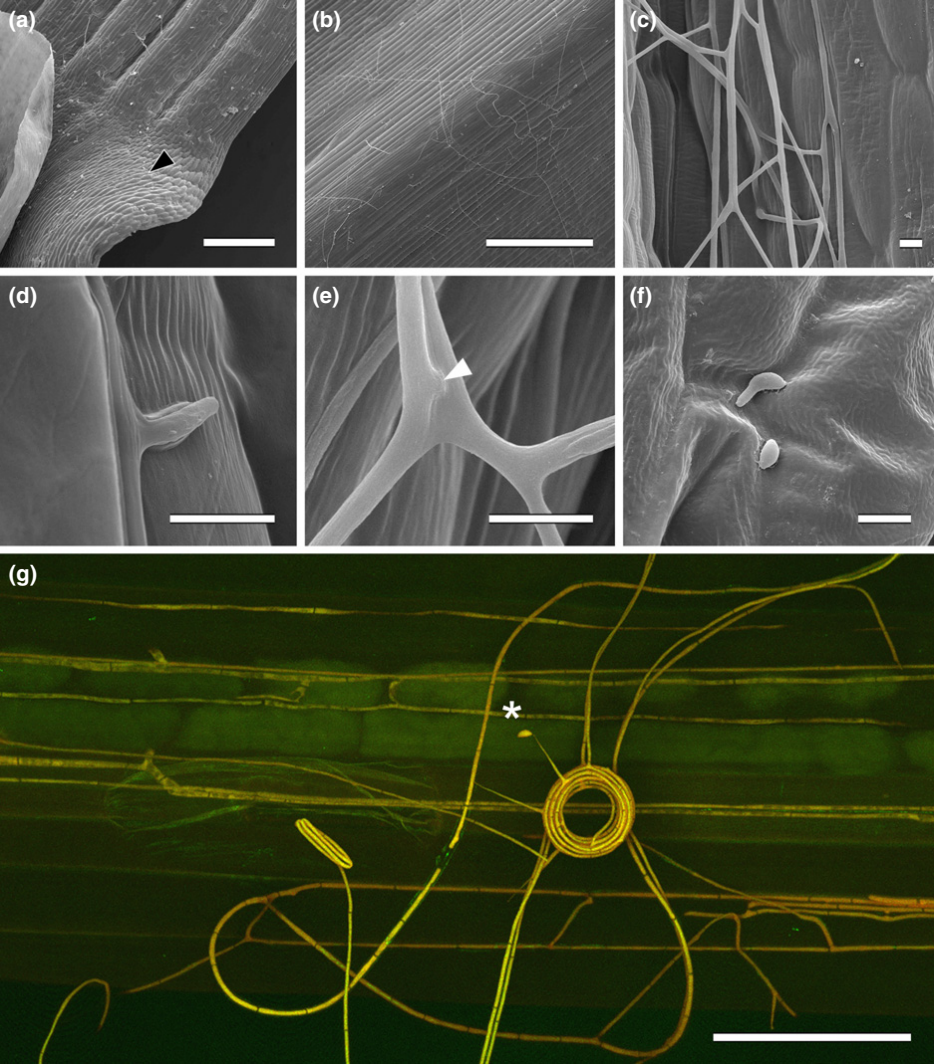
Epiphyllous growth of wild‐type (WT) *Epichloë festucae* on *Lolium perenne* leaf blade. (a) Endophytic hyphae on the adaxial surface of blade, close to the leaf expansion zone (black arrowhead). (b) Hyphae on abaxial blade surface. (c) Hyphal net in furrow of adaxial blade surface. (d) Hypha attached to plant surface by adhesive film. (e) Hyphal fusion (white arrowhead). (f) Germinating conidia. (g) Hyphal coil with conidiophore and conidium (asterisks). The confocal laser scanning microscopy image was generated by maximum intensity projection of *z*‐stacks. Bars: (a, b) 500 μm; (c–f) 10 μm; (g) 25 μm.

Scanning electron microscopy and CLSM analysis of leaves of *L. perenne* infected with *E. festucae* wild‐type strain Fl1 grown axenically under controlled environmental conditions showed epiphyllous hyphae on the surface of both sides of the leaves. The density of hyphae was greatest at the base of the leaf blade. The adaxial side of a grass leaf blade (Fig. 7a) is known to differ from the abaxial side (Fig. 7b) by its more heterogeneous cell composition and its undulating surface as a result of alternation of ridges and furrows. On the adaxial surface, epiphyllous hyphae were mostly attached to the leaf surface, growing along leaf surface depressions and frequently bridging the furrows (Fig. 7c). Hyphae frequently fused to form an epiphyllous net on the surface of the leaf (Fig. 7e). On the abaxial side, hyphae grow close to the leaf surface and appear to be firmly attached by an adhesive film, similar to that produced by hyphae growing inside the leaves (Fig. 7d) (Christensen *et al*., 2008). Germinating conidia were frequently seen on the leaf surface but no further differentiation was observed (Fig. 7f). A characteristic feature of epiphyllous nets of *E. festucae* is the formation of coiled structures (Fig. 7g) (Scott *et al*., 2012), which were also found in culture (Fig. S2). These structures result from hyphae coiling around on top of one another in one direction. The hyphal coils appear to be fully differentiated structures rather than intermediates in the formation of more complex structures such as fruiting bodies. Hyphal branching appeared to be more common in the hyphae comprising coils than in the surrounding hyphae, a developmental feature that would support expansion of hyphal nets. This mode of growth would allow *E. festucae* to rapidly cover large areas of the leaf surface. Conidiophores were also a common structural feature of coiled hyphae (Fig. 7g), suggesting that formation of coils is also a mechanism to increase the sporulation rate.

Hyphae on leaf sheaths were observed to make close contact with the surface (Fig. S3) and with one another (Fig. S3b–f). Hyphae that were in contact with surface depressions between longitudinal cells tended to follow those depressions (Fig. S3a), and, after some branching, to form a layer of parallel hyphae that were frequently fused (Fig. S3b,c). A similar mode of growth was observed for hyphae growing on the surface of the blade. However, while hyphae on the blade maintain close contact with the leaf surface, except where they bridge furrows, hyphae on the sheath were frequently detached from the surface and distorted, forming loops (Fig. S3a), torsions (Fig. S3e), and aggregations shortly after branching (Fig. S3f).

When *E. festucae* was grown on an agar surface, two different hyphal layers could be distinguished, one that extends from the centre of the colony to the edge, and another that differentiates on top of the first and is confined to the centre of the colony (Fig. S2a). The first layer was characterized by straight, unbranched hyphae, which were aligned parallel to one another and closely attached to the agar, providing colony access to nutrients (Fig. S2b). The second layer comprised hyphal bundles, growing on top of one another, with diameters of up to 30 μm, and containing up to 50 individual hyphae (Fig. S2c). These hyphae, together with nonaggregated highly branched hyphae, formed an aerial hyphal network that was characterized by coiled or beehive‐like structures of closely attached hyphae (Fig. S2d). The presence of these coil‐like structures was frequently associated with increased hyphal branching and formation of conidiophores, some of which had germinating conidia that potentially contribute to maturation of the second hyphal layer (Fig. S2f). The aggregated hyphae that comprise this layer had hyphal diameters varying from 0.5 to 2.0 μm (Fig. S2e).

### Plant blade phenotype of *E. festucae nox* mutants

Given the importance of both the Nox1 (= NoxA) and Nox2 (= NoxB) NADPH complexes for the differentiation of appressoria in *Magnaporthe oryzae* (Egan *et al*., 2007; Ryder *et al*., 2013) and our previous observation that the *E. festucae* ∆*noxA* mutant forms abundant epiphyllous growth (Tanaka *et al*., 2006), we analysed the role of NoxA, NoxB and NoxR on the development of expressoria in *E. festucae*. First we checked whole‐plant and cellular phenotypes of the *nox* mutants. Phenotypes were as previously reported (Takemoto *et al*., 2006; Tanaka *et al*., 2006), with the exception of the ∆*noxB* mutant (PN2469), which showed a disrupted symbiotic interaction phenotype under the controlled environment growth conditions used (Fig. 1). To confirm this phenotype, three new independent deletion mutants of *noxB* were generated (PN3062–PN3064) (Fig. S4); all had the same interaction phenotype as the original ∆*noxB* mutant.

Scanning electron microscopy revealed that the ∆*noxA,* ∆*noxAB* and ∆*noxR* mutants had increased epiphyllous growth compared with ∆*noxB* and the wild‐type (Fig. S5). We also observed that the epiphyllous growth phenotypes of the *nox* mutants on the blade surface were similar to that observed in culture under low nutrient conditions (Kayano *et al*., 2013). Sporulation of the ∆*noxA* and ∆*noxAB* mutants on the leaf surface was abundant (Fig. S6), results that recapitulate the conidiation phenotype of these mutants in culture (Kayano *et al*., 2013). However, there was considerable variability in the number of conidiophores observed among the leaf samples examined. In culture, ∆*noxA*, ∆*noxAB* and ∆*noxR* do not undergo cell–cell fusion, whereas fusion in the wild‐type and ∆*noxB* is frequently observed under low nutrient conditions (Kayano *et al*., 2013). Similarly, cell–cell fusions were observed for the wild‐type and the ∆*noxB* mutant growing on the surface of the leaf blade, but fusions were not observed for ∆*noxA*, ∆*noxAB* and ∆*noxR* (Fig. S7). However, these mutants still formed bundles of aggregated hyphae, as found in the wild‐type, which result in a pseudonet of epiphyllous hyphae on the surface (Fig. S7b,d,e).

**Figure 8:**
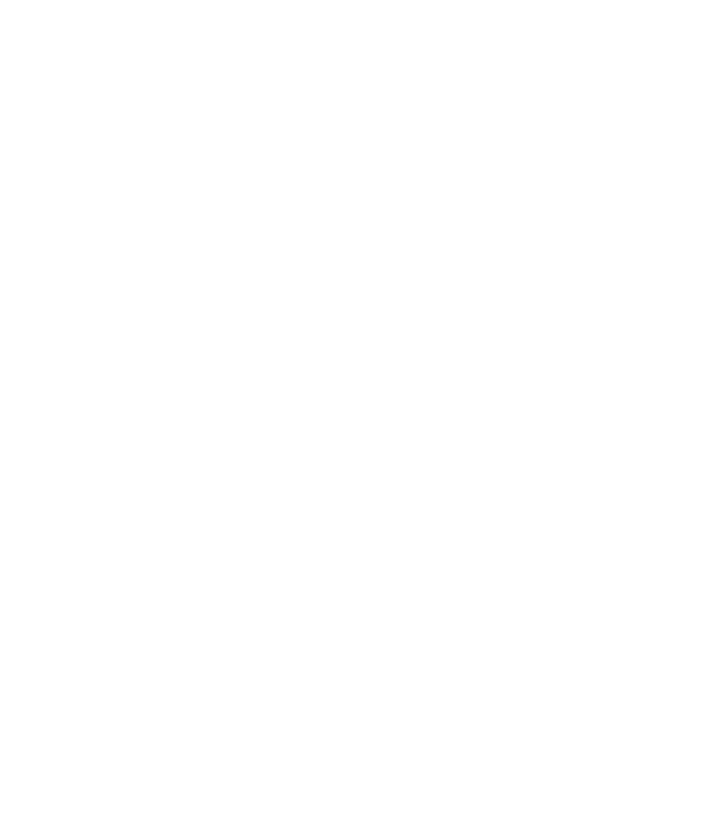
Scanning electron microscopy images showing subcuticular hyphal growth and cuticle breach of *Epichloë festucae noxA*,* noxB*,* noxAB*, and *noxR* mutants on *Lolium perenne* leaf blades. Black arrowheads point to subcuticular hyphae, black asterisks indicate points of hyphal emergence of subcuticular hypha, and white asterisks indicate points of direct hyphal emergence. Bars, 5 μm.

**Figure 9:**
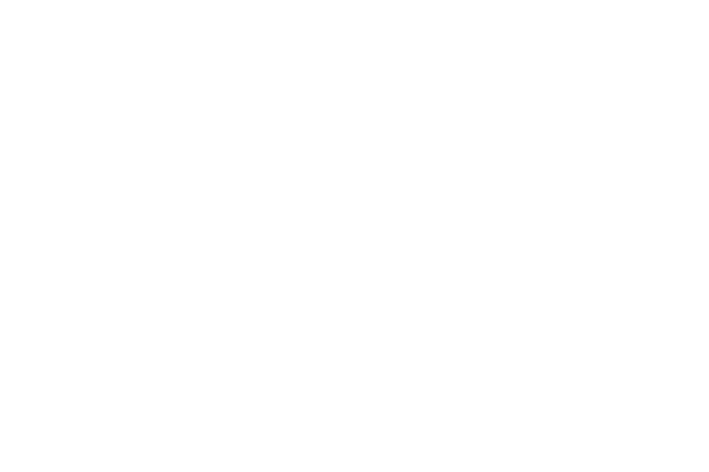
Subcuticular hyphae and cell wall changes of endophytic hyphae of the *Epichloë festucae* ∆*noxAB* mutant in *Lolium perenne*. (a) Endophytic hypha (en) not forming an expressorium on first contact with the cuticle (#) and forming hyphae of increased diameter in subsequent compartments while remaining below the cuticle (green pseudocolour). (b) Branched and swollen subcuticular hypha with septa captured in blue pseudocolour (WGA‐AF488 fluorescence). (c) Proliferation of subcuticular hyphae in a single epidermal cell. (d) Unusual branched and swollen pattern of a subcuticular hypha. WGA‐AF488 fluorescence indicative of chitin is shown in blue pseudocolour. (e) Subcuticular hypha with increased hyphal diameter in each new compartment. Also shown is a stoma (in green pseudocolour) and epiphyllous hyphae (cells entirely blue). (f, g) Higher magnifications of (e) showing uppermost layer of thickest hyphal compartment. (f) WGA‐AF488 fluorescence indicating the presence of chitin, and (g) ripped cuticle marked by white arrowheads. Maximum intensity projection *z*‐stacks of confocal laser scanning microscopy images of aniline blue/WGA‐AF488 costained leaf blade sample. Bars, 10 μm.

**Figure 10:**
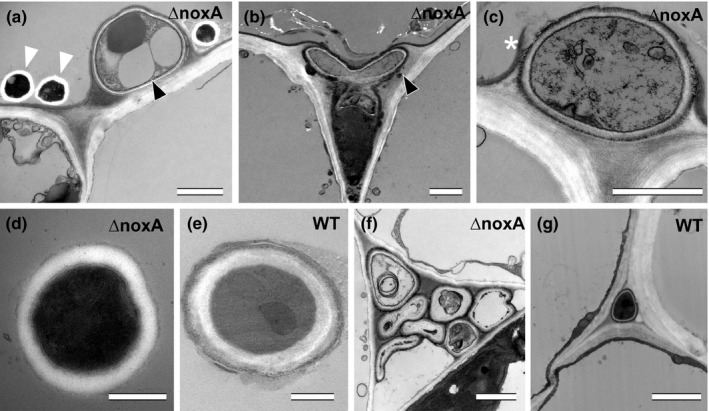
Ultrastructure of subcuticular hyphae, cuticle breaching, epiphyllous and endophytic hyphae of *Epichloë festucae* wild‐type (WT) and ∆*noxA* mutant in *Lolium perenne*. (a, b) Subcuticular growth of ∆*noxA* hyphae (black arrowhead) and epiphyllous hyphae (white arrowheads). (c) Cuticle breach of subcuticular hypha (white asterisk). (d, e) Ultrastructure of the cell wall of epiphyllous hyphae of WT and ∆*noxA* mutant. (f, g) Endophytic hyphae of WT and ∆*noxA* mutant. Transmission electron micrographs of WT and ∆*noxA* infected leaf blade cross‐sections near the ligule. Bars: (a–c, f, g) 2 μm; (d, e) 0.5 μm.

The most striking phenotype difference between wild‐type and the *nox* mutants was the abundance of subcuticular growth (Figs 3, 8). SEM analysis indicated that *nox* mutants seemed unable to immediately penetrate the cuticle, but instead continued to grow between cuticle and the outer cell wall of the epidermis (Fig. 8), a conclusion further supported by CLSM analysis of the ∆*noxAB* mutant (Fig. 9), and TEM (Fig. 10a,b) and CLSM (Fig. S8) analyses of the ∆*noxA* mutant. Subcuticular growth was occasionally seen in the wild‐type but is restricted to the leaf blade expansion zone, whereas in the ∆*noxA,* ∆*noxB* and ∆*noxAB* mutants, extensive subcuticular growth was observed along the length of the sheath and the blade. However, the extent of subcuticular growth varied from plant to plant, indicating that the growth response was plant genotype‐specific. In severely stunted plants, subcuticular hyphae were observed along the entire length of the sheath and blade (Fig. S8), whereas moderately stunted plants had fewer subcuticular hyphae, which might reflect the hyphal load in a single plant.

Another feature of the growth of these mutants was the presence of frequent hyphal branching in both the epidermal and subcuticular layers (Figs 9b,c, 10b) and abnormal branching (Fig. 9d), compared with the wild‐type. Subcuticular growth also appeared to be occasionally restricted to single epidermal cells (Fig. 9b,c), although growth to adjacent cells was also observed (Fig. S8). While subcuticular hyphae were thicker and epiphyllous hyphae thinner than endophytic hyphae in *nox* mutants (Fig. 3), the diameters of wild‐type endophytic and epiphyllous hyphae were similar. Wild‐type epiphyllous hyphae were significantly thicker than ∆*noxA*, ∆*noxB*, ∆*noxAB* and ∆*noxR* mutant epiphyllous hyphae (Figs 3, S9).

Subcuticular mutant hyphae were observed to eventually breach the cuticle (Figs 8, S10) and spread by unrestricted growth on the leaf surface to form an epiphyllous hyphal pseudonet. SEM (Fig. 8) and TEM (Fig. 10c) analyses suggest that direct breach of the cuticle following subcuticular growth is the most common exit mechanism, responsible for the abundance of epiphyllous hyphae in the *nox* mutants. However, *nox* mutants still formed hyphal swellings, but they were frequently misshapen and failed to polarize at the point of contact with the cuticle to form penetrating hyphae (Fig. 8).

**Figure 11:**
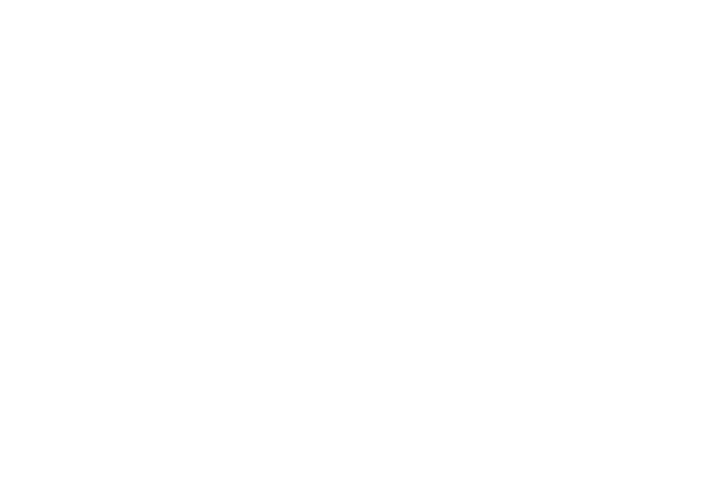
Cell wall changes in endophytic hyphae of *Epichloë festucae* ∆*noxR* mutant in *Lolium perenne*. (a–c) Abundance of WGA‐AF488 fluorescence, indicative of chitin in swollen compartments (diameter up to 8 μm) of endophytic hyphae (asterisk); the black arrowhead points to an aniline blue‐stained cell wall layer. (d) An intrahyphal hypha (WGA‐AF488 fluorescence captured in blue pseudocolour) growing inside a heavily degraded endophytic hypha (stained with aniline blue, fluorescence captured in orange pseudocolour) and exiting from degraded hypha (#). (e) Abundance of WGA‐AF488 fluorescence in two hyphae below stoma surrounded by an aniline blue‐stained cell wall layer (black arrowheads). (f, h) Higher magnifications of (g) showing highly differentiated leaf blade tissue with large intercellular spaces. (f) WGA‐AF488 fluorescence at tips of endophytic hyphae below open stoma. (h) Side view of hypha penetrating between epidermal cells and exiting the plant (epiphyllous hypha; ep); the white arrowhead marks plant wound response where the hypha enters the area of middle lamella between epidermal cells. Maximum intensity projection *z*‐stacks of confocal laser scanning microscopy images of aniline blue/WGA‐AF488 costained leaf blade sample. Bars, 10 μm.

As observed for the wild‐type, the *nox* mutants also underwent a major remodelling of the cell wall when they transitioned from endophytic to epiphyllous growth. CLSM of WGA‐AF488‐stained tissue infected with the ∆*noxAB* mutant showed that remodelling of the cell wall in subcuticular hyphae started when the cuticle was ruptured (Fig. 9e–g). However, there was also some remodelling of the cell wall in endophytic hyphae of the *nox* mutants as evidenced by the WGA‐AF488 fluorescence observed in sections of the cell walls for the ∆*noxR* (Fig. 11). Altered patterns of chitin distribution were observed for endophytic hyphae growing in the mesophyll (Fig. 11a–c) and near stomata (Fig. 11e,f). By contrast, just the septa of wild‐type endophytic hyphae fluoresced. Another phenotype observed for the *nox* mutants was formation of intrahyphal hyphae. Interestingly, the cell walls of these inner hyphae fluoresced with WGA‐AF488 (blue pseudocolour), whereas the outer hyphae did not, but still fluoresced with aniline blue (orange/red pseudocolour) (Fig. 11d). The WGA‐AF488 signal remained stable even when the intrahyphal hyphae broke through the enveloping hyphae.

**Figure 12:**
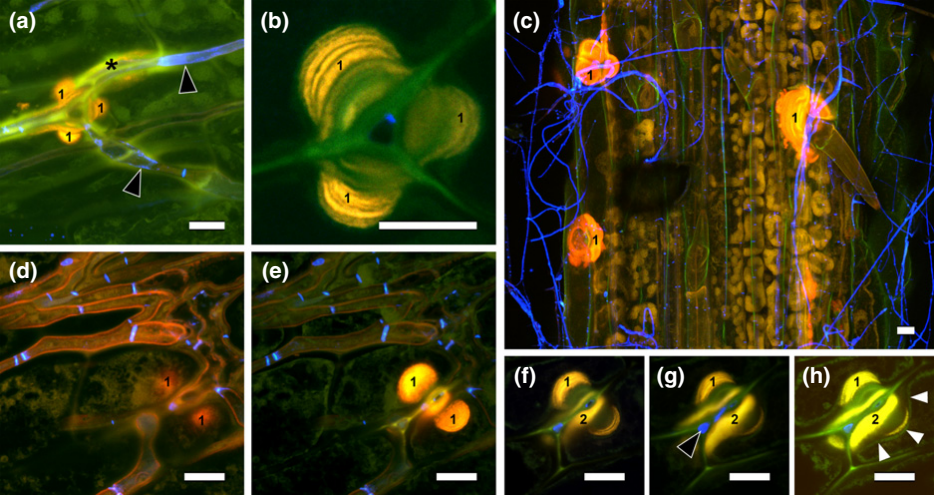
Wound response of *Lolium perenne* to *Epichloë festucae* ∆*noxA* mutant. (a) WGA‐AF488 fluorescence indicative of chitin (black arrow heads) in endophytic hyphae and plant autofluorescence (yellow) indicative of phenolic compounds in anticlinal epidermal cell walls (‘1’) as well as wound response between the mesophyll and epidermis (asterisk). (b) Enlargement of (a) showing several layers of cell wall deposition (probably cellulose and lignin). (c) Numerous wound responses in anticlinal epidermal cell walls and cell wall depositions (‘1’). (d–g) Ascending substacks of an endophytic hypha penetrating between epidermal cells showing plant autofluorescence indicative of a plant wound response in the anticlinal epidermal cell walls close to mesophyll (‘1’) and on top of the periclinal epidermal cell walls (‘2’). (h) Overexposed image from *z*‐stack of (d–g), highlighting the plasmalemma (white arrowheads) indented by plant cell wall thickening. Confocal laser scanning microscopy images of aniline blue/WGA‐AF488 costained blade sample. Bars, 10 μm.

No response of the plant to hyphal exit was observed for the wild‐type (Figs 5, 6). However, the exit of *nox*‐mutant hyphae induced two different plant wound/defense responses, as visualized by the yellow autofluorescence of the plant tissue (Figs 12, S11). The first response observed was a thickening of the plant cell walls, possibly fluorescing as a result of incorporation of lignin, when hyphae were growing between anticlinal epidermal cell walls (Fig. 12a–e; #1). The second response was formation of callose when hyphae ruptured the cuticle (Fig. 12f–h). This CLSM analysis was supported by TEM analysis. An electron‐dense layer, which is possibly callose, was observed adjacent to a ∆*noxA* hypha (Fig. S11e).

## Discussion

Establishment of symbiotic associations between fungi and plants relies on the ability of the fungus to colonize the host. Many pathogenic fungi have specialized structures, such as appressoria, to breach the physical barriers of the plant host (Emmett & Parbery, 1975; Tucker & Talbot, 2001) or alternatively they colonize through natural plant openings such as stomata or through wounds. By contrast, *E. festucae* is transferred to new host plants predominantly by vertical transmission through the seed, resulting in asymptomatic associations with host plants (Leuchtmann *et al*., 1994; Schardl, 2001). However, as part of the sexual life cycle, ascospores of *E. festucae* can be transmitted horizontally to the florets of a new host plant where they germinate to form infectious hyphae that colonize the ovary and developing seed (Schardl, 2001) by a mechanism similar to that used by *Claviceps purpurea* to colonize rye (Chung & Schardl, 1997; Tudzynski & Tenberge, 2003; Tudzynski & Scheffer, 2004). After seedling development, *E. festucae* systemically colonizes the intercellular spaces of aerial tissues of the plant by a combination of both tip and intercalary growth, to establish a symbiotic association with the host (Christensen *et al*., 2008; Scott *et al*., 2012). As a consequence of this known biology, there is no obvious need for *E. festucae* to develop specialized host infection structures. However, in this study we have identified a novel, appressorium‐like structure, which we propose to call an expressorium, which allows endophytic hyphae of *E. festucae* to breach the cuticle of the leaf to form an epiphyllous hyphal net on the leaf surface. Although this structure appears to be very different to classic appressoria (Emmett & Parbery, 1975), differentiation of *E. festucae* expressoria requires both NoxA and NoxB complexes, suggesting that the underlying mechanism for leaf exit may be similar to that of appressoria‐mediated leaf entry by *M. oryzae* (Egan *et al*., 2007; Ryder *et al*., 2013) and other plant pathogenic fungi (Giesbert *et al*., 2008; Segmüller *et al*., 2008). The ability of *E. festucae* to form an expressorium allows this fungus to form an interconnected hyphal network in the grass host that comprises both endophytic and epiphytic hyphae, highlighting the complex nature of *E. festucae* when growing in symbiotic association with its grass host.

Appressoria are distinct fungal infection structures for plant host invasion and are usually derived from germ tubes or hyphal apices following contact with a solid surface (reviewed by Emmett & Parbery, 1975). Both thigmotropic and chemotropic signals are important for differentiation of these structures on leaf surfaces (reviewed by Dean, 1997; Skamnioti & Gurr, 2007). Although we generally associate appressoria formation with epiphytic hyphae, they can also arise from the apices of endophytic hyphae to produce infection pegs that allow hyphae to pass from cell to cell (reviewed by Emmett & Parbery, 1975). While there are some similarities between appressoria and expressoria, such as the formation of swollen structures, the morphogenesis and development of the latter appear to be fundamentally different. Expressoria arise immediately below the cuticle from apices of hyphae that emerge from between tightly packed epidermal cells. Therefore, they have just the cuticle to breach to gain access to the surface of the leaf, whereas appressoria must generate sufficient turgor pressure to penetrate both the cuticle and epidermal cell to infect the host plant (Emmett & Parbery, 1975; Tucker & Talbot, 2001). Contact of *E. festucae* endophytic hyphae with the cuticle triggers formation of hyphal swellings that are demarcated by septa on both sides. The pressure exerted by expressoria leads to a thinning of the cuticle layer and eventual breach of this physical barrier. Whether breakage of this layer is just a physical process or involves enzymes such as cutinases is not known; *E. festucae* does have four genes of the CAZy carbohydrate esterase class CE5 (cutinases), two (EfM3.008610 and EfM3.037435) of which are up‐regulated in symbiosis mutants that undergo proliferative growth instead of restrictive growth *in planta* (Eaton *et al*., 2015). While we could not find any evidence for a penetration peg, expressoria do repolarize at the site of cuticle thinning to form new hyphal apices that emerge on the surface of the leaf, where they appear to undergo a thigmotropic response to bend and reorientate their growth along the surface of the leaf.

Repolarization of the *E. festucae* expressorium at the site of leaf emergence requires NoxA, NoxB and NoxR. The most distinctive phenotype of all of these mutants is the abundance of subcuticular hyphae. Although contact with the cuticle appears still to trigger formation of hyphal swellings in endophytic hyphae of the *nox* mutants, they are frequently misshapen and branched, and fail to polarize at the point of contact with the cuticle. The need for both NoxA and NoxB complexes for expressorium development in *E. festucae* suggests that development of this swollen structure may be similar to appressorium development in *M. oryzae*, where the sequential action of Nox2 and Nox1 complexes is required for differentiation (Ryder *et al*., 2013). The Nox2 complex is required in the first step for septin‐mediated F‐actin reorientation at the appressorium pore, and the Nox1 complex is required at the second step for maintenance of the cortical F‐actin network during hypha elongation and host cell penetration (Dagdas *et al*., 2012; Ryder *et al*., 2013). The apparent generation of turgor pressure by the expressorium at the cuticle interface is another feature in common (Bourett & Howard, 1990; deJong *et al*., 1997).

Although the hyphae of the *nox* mutants are impaired in the development of expressoria, they do eventually breach the cuticle to emerge and proliferate on the surface of the leaf. The abundance of epiphyllous hyphae probably reflects the fact that mutations in the Nox complex components generate strains that undergo proliferative tip growth instead of restrictive intercalary growth, which characterizes the wild‐type interaction (Christensen *et al*., 1997; Tanaka *et al*., 2006; Christensen & Voissey, 2007; Scott *et al*., 2012). The increased expression of genes encoding cell wall‐degrading and hydrolytic enzymes by the *noxA* mutant suggests that the physiology of these mutants is very different from the wild‐type (Eaton *et al*., 2015). This switch to proliferative growth may be triggered by a starvation response as a result of the inability of the *noxA* and *noxR* mutants to undergo cell–cell fusion (Becker *et al*., 2015; Eaton *et al*., 2015). The cell–cell fusion phenotypes of the *nox* mutants, as determined by SEM analysis of the hyphae growing on the leaf surface, were identical to that determined by growth in axenic culture; that is, Δ*noxA*, Δ*noxAB* and Δ*noxR* were all defective in cell–cell fusion, whereas Δ*noxB* fused to the same extent as the wild‐type strain (Kayano *et al*., 2013).

Although epiphyllous hyphae of Δ*noxA* and Δ*noxAB* produce abundant conidiophores and conidia, we did not see in either wild‐type or nox mutants any appressoria‐like structures on the leaf surface, suggesting that contact to the outside of the cuticle is not sufficient for triggering formation of hyphal swellings and that *E. festucae* has developed a mechanism for exit from, but not entry into, *L. perenne* leaves. A possible explanation for this specificity could be differences in the signalling between the host and fungal symbiont on the outside and the inside of the leaf. Key cues for appressorium differentiation and host invasion in phytopathogenic fungi are surface hydrophobicity and cutin monomers (Tucker & Talbot, 2001; Kumamoto, 2008). Sensing of these signals at the membrane surface occurs through the mucin‐like protein Msb2 and the tetraspan protein Sho1 in the plant pathogenic fungi *Ustilago maydis* (Lanver *et al*., 2010), *M. oryzae* (Dixon *et al*., 1999; Liu *et al*., 2011), and *Fusarium oxysporum* (Pérez‐Nadales & Di Pietro, 2011) with subsequent transduction of the signal through conserved cAMP and MAP kinase signalling pathways (Lee & Dean, 1993; Xu & Hamer, 1996; Choi & Dean, 1997; Zhao *et al*., 2005; Doehlemann *et al*., 2006). It will be interesting to test whether the *E. festucae* homologue of Msb2 is also crucial for expressorium development on the inside of an *L. perenne* leaf in the blade and sheath intercalary cell division zones.

The other dramatic difference between endophytic and epiphytic hyphae is the structure and composition of the cell walls, as revealed by imaging leaf tissue infiltrated with WGA‐AF488 and by TEM. Wheat germ agglutinin binds to sialic acid and *N*‐acetylglucosamine residues, and is commonly used as an indicator for chitin (Robin *et al*., 1986). In wild‐type endophytic hyphae, only the septa fluoresce, whereas the entire cell wall of epiphytic hyphae fluoresce, suggesting that cell wall chitin is either masked or inaccessible to this lectin in the former but readily accessible in the latter. Interestingly, remodelling of the hyphal cell wall does not take place immediately after hyphae breach the cuticle, but becomes visible after two to four cell divisions. This observation and the fact that the entire cell wall of intrahyphal hyphae of the Δ*noxA* mutant fluoresces provide good evidence that the absence of cell wall fluorescence of endophytic hyphae of the wild‐type strain is not a result of a lack of infiltration of WGA‐AF488. There are several possible explanations for the apparent absence of chitin in the cell wall of the endophytic hyphae (including the expressorium): chitin is indeed absent; chitin is masked by other components of the fungal cell wall, such as α‐1,3‐glucan (Fujikawa *et al*., 2009, 2012), but as previously discussed, *E. festucae* appears to lack a homologue of a gene encoding an α‐1,3‐glucan synthase (Becker *et al*., 2015); chitin is masked by *E. festucae* LysM effector proteins similar to Avr4 and Ecp6 found in *Cladosporium fulvum* (van den Burg *et al*., 2006; van Esse *et al*., 2007; de Jonge *et al*., 2010); chitin is converted by a chitin de‐*N*‐acetylase(s) to chitosan, a form of chitin that is not recognized by WGA‐AF488, as occurs in the cell walls of infection structures of a number of plant pathogenic fungi, including *Puccinia graminis, Uromyces fabae* and *Colletotrichum graminicola* (El Gueddari *et al*., 2002). Whatever is the correct mechanism, the structure and composition of the hyphal cell wall appear to be very important for maintaining a mutualistic symbiotic interaction.

Interestingly, no host defence response appeared to be induced in the young undifferentiated cells of the sheath and blade intercalary division zones where expressoria of the wild‐type strain emerge. By contrast, two types of cell defence responses were observed for the *nox* mutants as visualized by an increase in autofluorescence: cell wall thickening and an increase in callose deposition. The differences in response are probably linked to the inability of *nox* mutants to form true expressoria, which gives the plant more time to respond once hyphae have reached the subcuticular layer. However, the host defence response observed for *nox* mutants may also reflect the fact that the *nox* mutants are able to breach the cuticle in mature leaf tissue because of their altered physiology as supported by the dramatic differences in the transcriptome of the *noxA* mutant compared with the wild‐type (Eaton *et al*., 2015). Either the nascent epidermal cells are unable to elicit an innate immunity response or, alternatively, *E. festucae* secretes effectors in this young tissue that suppress innate immunity by a mechanism similar to that of a lipase effector of *Fusarium graminearum* (Blümke *et al*., 2014). In support of the latter hypothesis, host genes for callose synthesis are down‐regulated and genes for callose degradation are up‐regulated in the host transcriptome of wild‐type infected plants compared with uninfected plants (Dupont *et al*., 2015). These subtle but important differences in the host interaction phenotype highlight the very fine balance between a mutualistic and antagonistic symbiotic interaction between *E. festucae* and *L. perenne*.

In conclusion, we demonstrate here that the interaction of *E. festucae* with its host *L. perenne* involves both endophytic and epiphytic hyphae and that differentiation of an expressorium below the cuticle of the leaf blade and sheath intercalary division zones is an important developmental step in allowing symptomless penetration of the cuticle and formation of an epiphyllous hyphal net on the surface of the leaf. Crucially, both the NoxA and NoxB complexes are required for this multicellular differentiation step. Identifying the mechanisms that regulate the changes in the cell wall associated with these two symbiotic states, and how *E. festucae* avoids triggering an innate immunity response in the host, will be important areas of research for future study.

## Author contributions

M.B., Y.B. and B.S. planned and designed the research. M.B., Y.B. and K.G. performed the experiments. M.B., Y.B. and B.S. analysed the results. M.B., Y.B. and B.S. wrote the manuscript.

## Supporting information

Please note: Wiley Blackwell are not responsible for the content or functionality of any supporting information supplied by the authors. Any queries (other than missing material) should be directed to the *New Phytologist* Central Office.

**Fig. S1** Growth of *E. festucae* wild‐type around the shoot apical meristem, in the leaf blade expansion zone of *L. perenne*, on the leaf surface and between epidermis and mesophyll.
**Fig. S2** *E. festucae* wild‐type culture phenotype on PD agar.
**Fig. S3** Epiphyllous growth of wild‐type *E. festucae* hyphae on *L. perenne* leaf sheath.
**Fig. S4** Deletion of *E. festucae noxB*.
**Fig. S5** SEM images showing epiphyllous hyphae of *E. festucae* wild‐type and ∆*noxA*, ∆*noxB,* ∆*noxAB* and ∆*noxR* mutants on the adaxial side of *L. perenne* leaf blades.
**Fig. S6** SEM images showing asexual sporulation of epiphyllous hyphae of *E. festucae* wild‐type and *noxA*,* noxB, noxAB*,* noxR* mutants on *L. perenne* leaf blades.
**Fig. S7** SEM images showing hyphal cell–cell fusion of epiphyllous hyphae of *E. festucae* wild‐type and *noxA*,* noxB, noxAB*,* noxR* mutants on *L. perenne* leaf blades.
**Fig. S8** Epiphyllous hyphae of *E. festucae* wild‐type compared with subcuticular hyphal growth of the ∆*noxA* mutant on *L. perenne* leaf sheath and blade.
**Fig. S9** SEM images of epiphyllous growth of *nox* mutants of *E. festucae* on adaxial blade surface of *L. perenne*.
**Fig. S10** Subcuticular hyphal growth and cuticle breaching of *E. festucae* ∆*noxA* on *L. perenne* leaf blade.
**Fig. S11** Wound response of *L. perenne* to the presence of *E. festucae* ∆*noxAB* mutant.
**Table S1** Strains and plasmids used in this study
**Table S2** Primers used in this study

**Movie S1** Movie capturing images taken in the *z* plane of epiphyllous hyphae and expressorium of wild‐type *E. festucae* in symbiosis with *L. perenne* presented in Fig. 2(c).

**Movie S2** Movie capturing images taken in the *z* plane of an expressorium of wild‐type *E. festucae* in symbiosis with *L. perenne* penetrating the host cuticle.

**Movie S3** Movie capturing images taken in the *z* plane of epiphyllous hyphae and expressorium of wild‐type *E. festucae* in symbiosis with *L. perenne*.

**Movie S4** Movie capturing images taken in the *z* plane of epiphyllous hyphae and expressorium of wild‐type *E. festucae* in symbiosis with *L. perenne* presented in Fig. 4(b).
