# Supplementary material for: The endophytic symbiont Epichloë festucae establishes an epiphyllous net on the surface of Lolium perenne leaves by development of an expressorium, an appressorium‐like leaf exit structure
Source: New Phytol. 2016 Mar 17;211(1):240–54. doi: 10.1111/nph.13931 (PMC5069595; doi:10.1111/nph.13931)
Supplement: Supplementary file 1 — Fig. S1 Growth of E. festucae wild‐type around the shoot apical meristem, in the leaf blade expansion zone of L. perenne, on the leaf surface and between epidermis and mesophyll. Fig. S2 E. festucae wild‐type culture phenotype on PD agar. Fig. S3 Epiphyllous growth of wild‐type E. festucae hyphae on L. perenne leaf sheath. Fig. S4 Deletion of E. festucae noxB. Fig. S5 SEM images showing epiphyllous hyphae of E. festucae wild‐type and ∆noxA, ∆noxB, ∆noxAB and ∆noxR mutants on the adaxial side of L. perenne leaf blades. Fig. S6 SEM images showing asexual sporulation of epiphyllous hyphae of E. festucae wild‐type and noxA, noxB, noxAB, noxR mutants on L. perenne leaf blades. Fig. S7 SEM images showing hyphal cell–cell fusion of epiphyllous hyphae of E. festucae wild‐type and noxA, noxB, noxAB, noxR mutants on L. perenne leaf blades. Fig. S8 Epiphyllous hyphae of E. festucae wild‐type compared with subcuticular hyphal growth of the ∆noxA mutant on L. perenne leaf sheath and blade. Fig. S9 SEM images of epiphyllous growth of nox mutants of E. festucae on adaxial blade surface of L. perenne. Fig. S10 Subcuticular hyphal growth and cuticle breaching of E. festucae ∆noxA on L. perenne leaf blade. Fig. S11 Wound response of L. perenne to the presence of E. festucae ∆noxAB mutant. Table S1 Strains and plasmids used in this study Table S2 Primers used in this study [file NPH-211-240-s001.pdf]

New Phytologist

***New Phytologist* Supporting Information**

Article title: **The endophytic symbiont *Epichloë festucae* establishes an epiphyllous net on the surface of *Lolium perenne* leaves by development of an expressorium, an appressorium-like leaf exit structure**

Authors: Matthias Becker, Yvonne Becker, Kimberly Green and Barry Scott

Article acceptance date: 07 February 2016

The following Supporting Information is available for this article:

Fig. S1 Growth of *E. festucae* wild-type around the shoot apical meristem, within the leaf expansion zone in mesophyll and epidermis and on leaf surface of *L. perenne*. (a) Scanning electron micrograph of hyphae growing in the vicinity of the SAM. Black arrowheads identify hypha emerging from developing leaf blades. (b–d) CLSM series of descending z-stacks showing growth of young epiphyllous hyphae (ep) after emergence from the cuticle on the leaf surface (b), endophytic hyphae (en) in epidermal cell layer (c), and in mesophyll tissue (d). Bars: (a) 100 µm; (b–d) 10 µm.

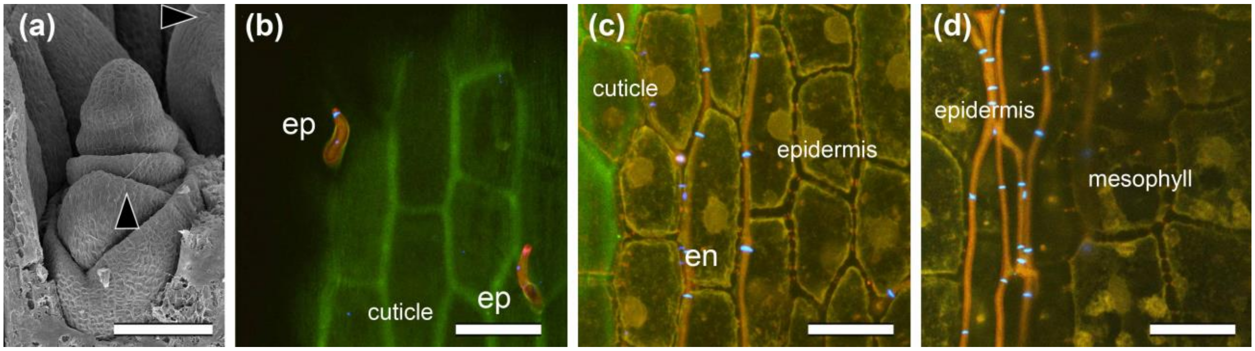

New Phytologist

Fig. S2 *E. festucae* wild-type culture phenotype on PD agar. (a) Image of the colony from centre to periphery of plate. (b) Hyphal bundles on peripheral edge of colony showing attachment to the agar. (c) Hyphal bundles and aerial hyphae from central region of colony. (d) Hyphal coil. (e) Hyphal swelling. (f) A germinating spore attached to a conidiophore. Scanning electron microscopy (SEM) images of 10-d-old cultures. Bars: (a) 100 μm; (b–d) 10 μm; (e, f) 5 μm.

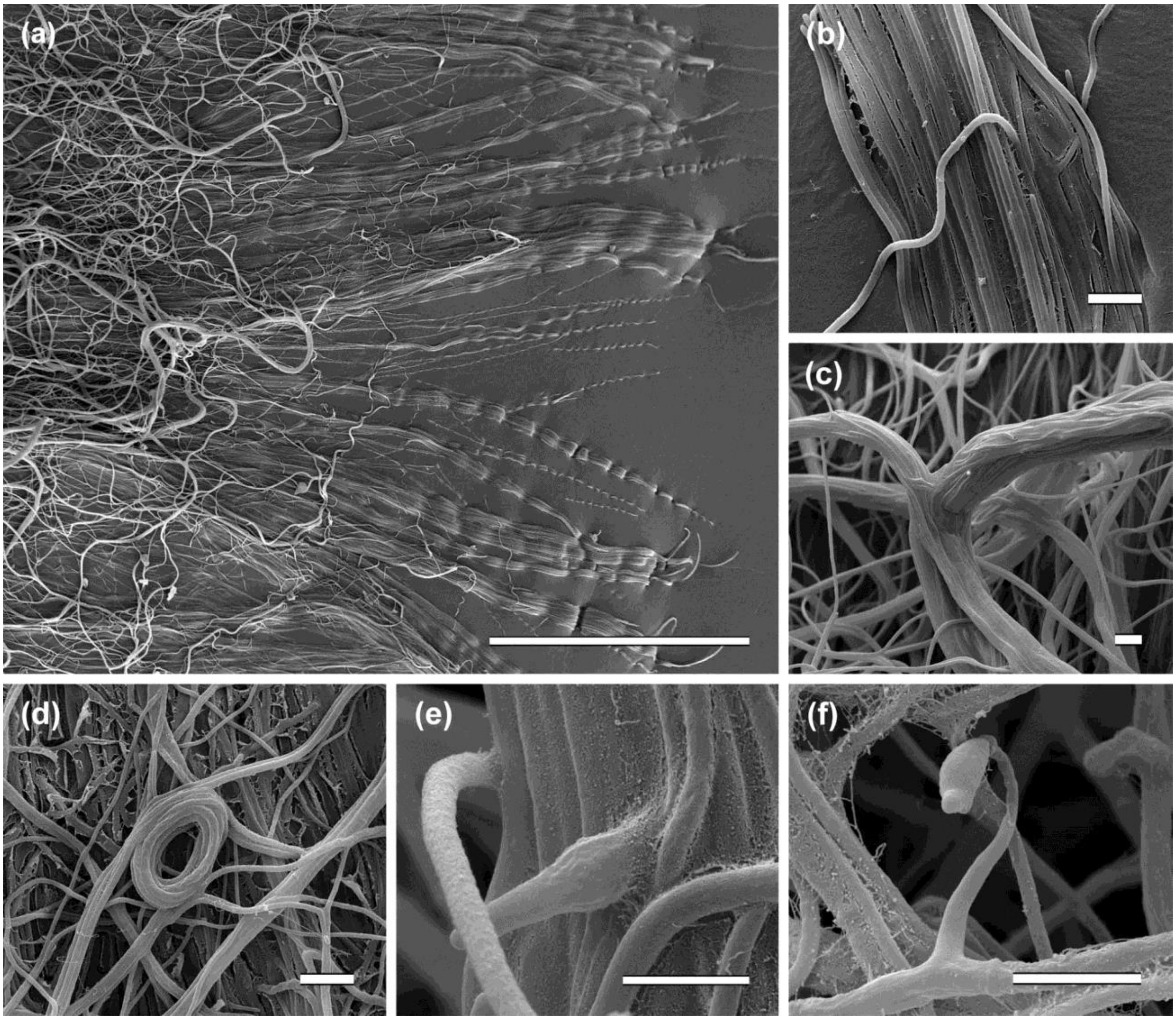

Fig. S3 Epiphyllous growth of wild-type *E. festucae* hyphae on *L. perenne* leaf sheath. (a) Hypha emerging from between epidermal cells (asterisks) and distorted hyphal growth until hypha establishes contact with the leaf in the depression between plant cells (black arrowhead). (b) Hyphal bundles and (c) hyphal cell–cell fusion (white arrowheads). (d) Hypha branches after emergence from between epidermal cells (asterisk). (e) Distorted hyphal growth with hyphae winding around each other and (f) branching. Images were captured using scanning electron microscopy (SEM). Bars, 10 μm.

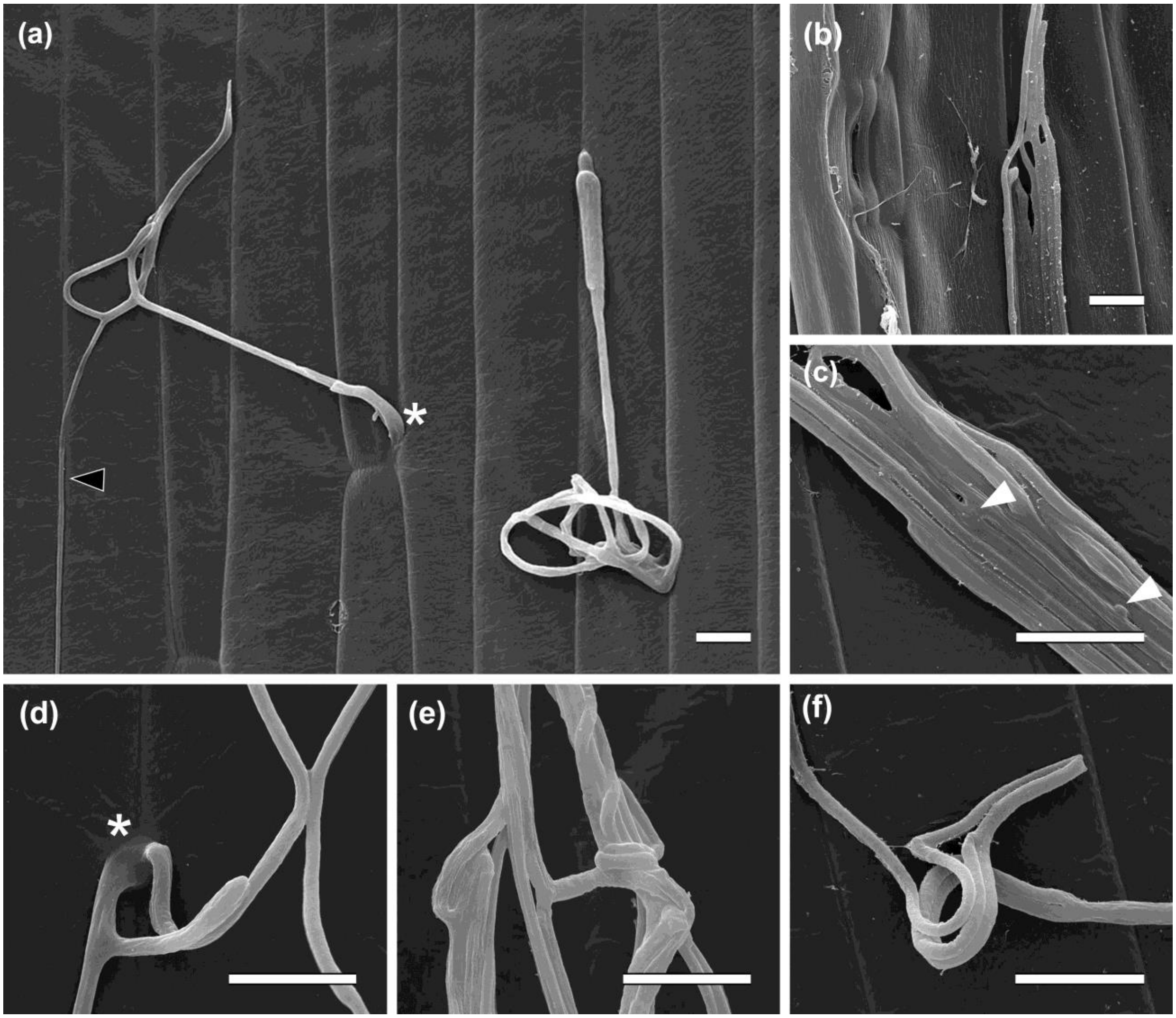

Fig. S4 Deletion of *E. festucae noxB*. (a) Physical map of the wild-type (WT) *noxB* genomic region, linear component of the replacement construct pPN78, the Δ*noxB* mutant genomic region and corresponding complementation vector. Regions of homology between the replacement construct and the wild-type genome are indicated by grey shading. Primers used to amplify the replacement fragment of pPN78 (168/169), to screen for transformants (170/165 and 166/171), to amplify the pSF15.15 vector backbone (180/181) and *noxB* fragment (178/179), are marked by arrowheads. (b) Autoradiograph of a Southern blot of *Sac*I genomic digests of WT and Δ*noxB* strains probed with DIG-labelled PCR fragment (168/169).

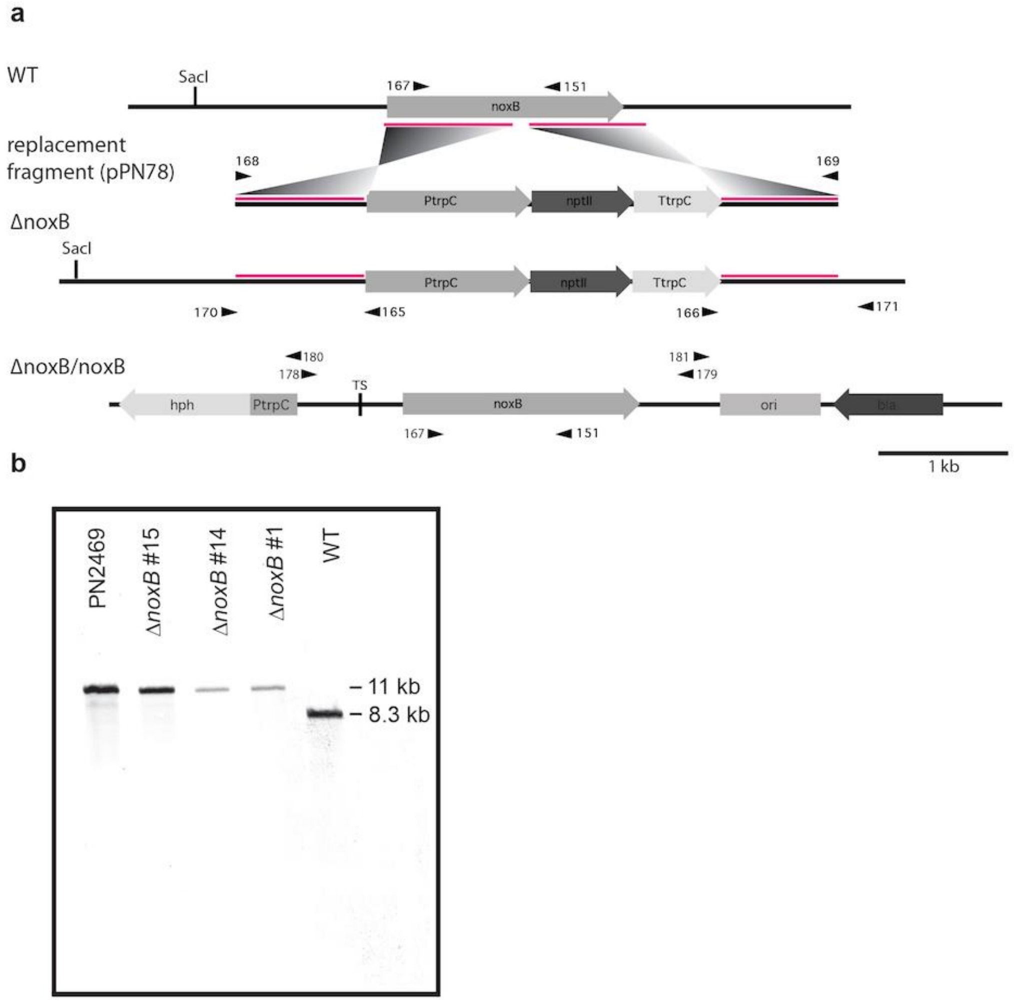

Fig. S5 SEM images showing epiphyllous hyphae of *E. festucae* wild-type and *noxA*, *noxB*, *noxAB*, *noxR* mutants on the adaxial side of *L. perenne* leaf blades. Note, that the ligule has been ripped off during preparation to allow view of leaf blade intercalary extension zone. (a) WT, (b) Δ*noxA*, (c) Δ*noxB*, (d) Δ*noxAB*, (e) Δ*noxR*. Bars, 200 μm. These are representative images of the leaf colonization phenotype observed from a multiple images.

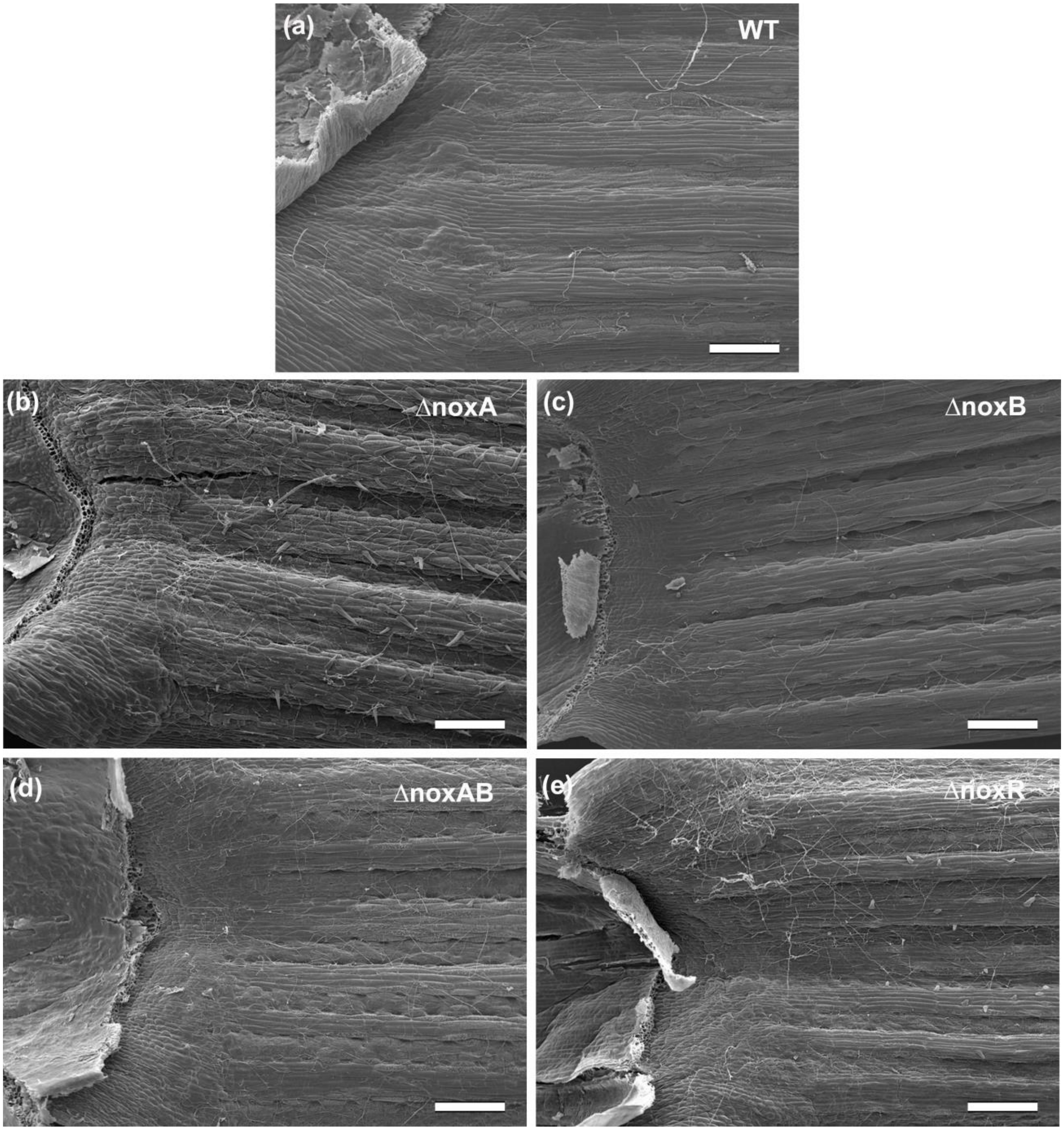

Fig. S6 SEM images showing asexual sporulation of epiphyllous hyphae of *E. festucae* wild-type and *noxA, noxB, noxAB, noxR* mutants on *L. perenne* leaf blades. Images show conidiophores capped with single conidia growing from epiphyllous hyphae (a) WT, (b, c) Δ*noxA*, (d) Δ*noxB*, (e) Δ*noxAB*, (f) Δ*noxR*. Bars, 10 µm.

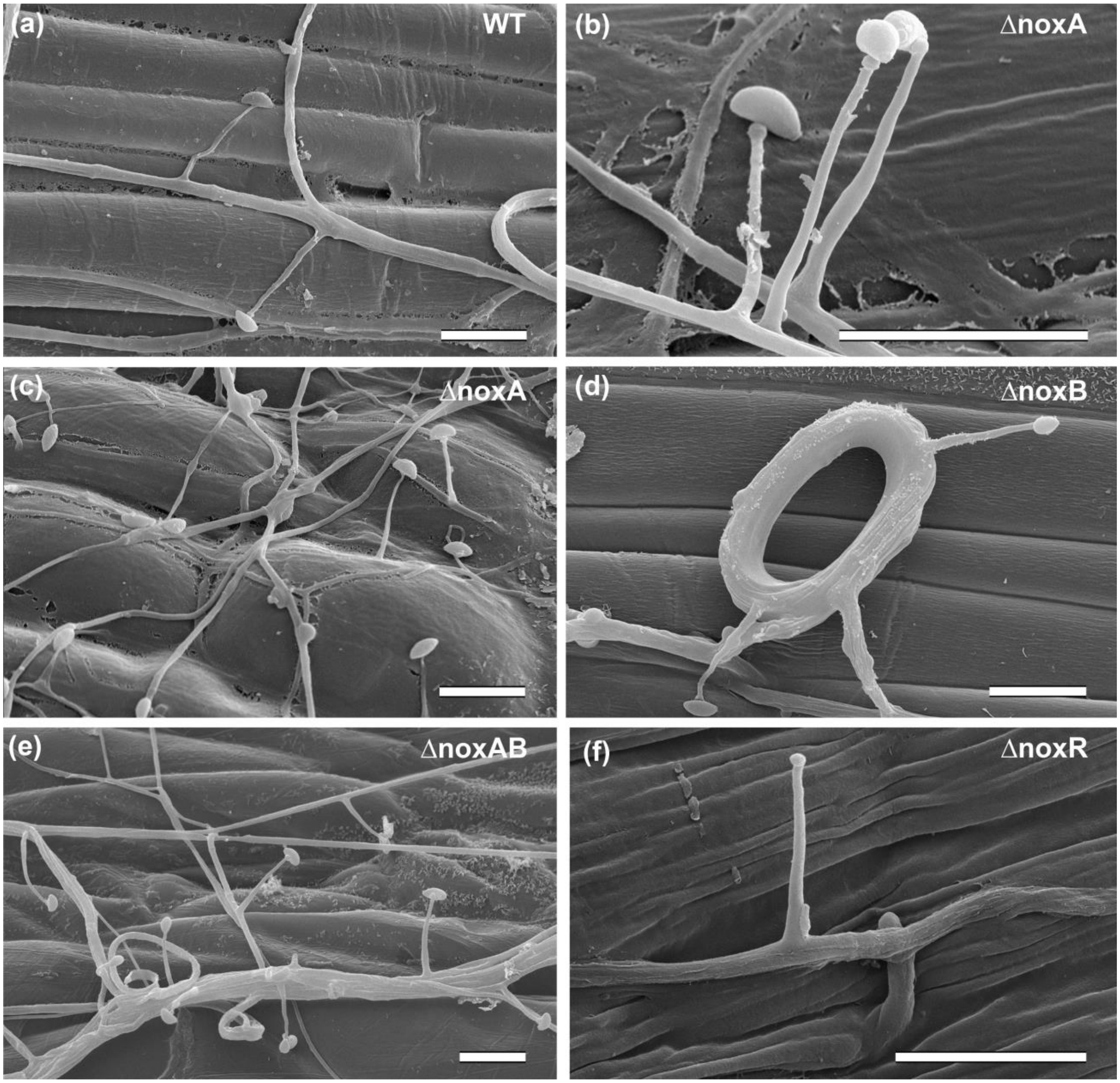

New Phytologist

Fig. S7 SEM images showing hyphal cell–cell fusion of epiphyllous hyphae of *E. festucae* wild-type and *noxA*, *noxB, noxAB*, *noxR* mutants on *L. perenne* leaf blades. (a) WT, (b) Δ*noxA*, (c) Δ*noxB*, (d) Δ*noxAB* and (e) Δ*noxR*. White arrowheads point to fusion sites (a, c). Bars, 1 µm.

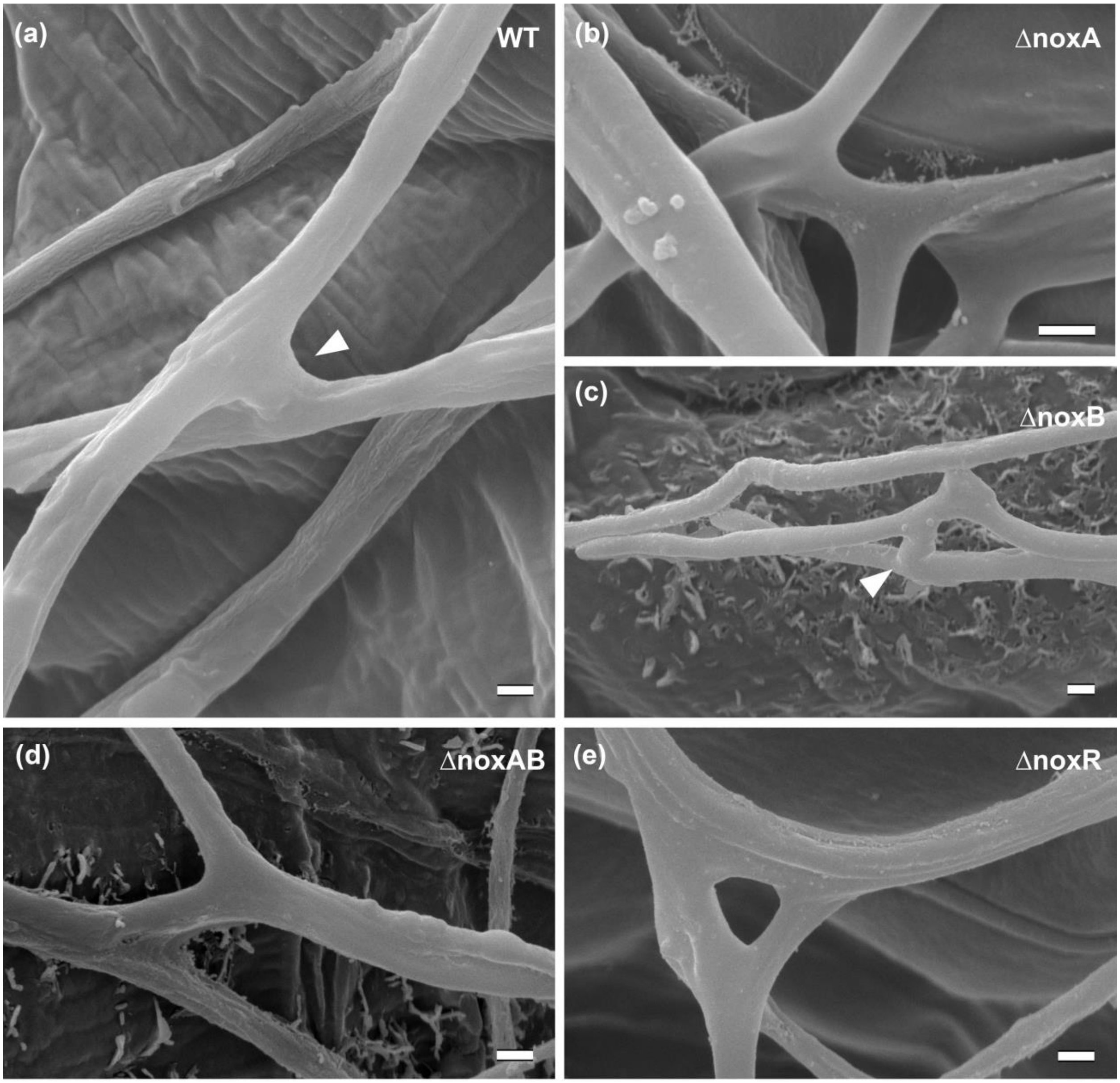

Fig. S8 Epiphyllous hyphae (ep) of *E. festucae* wild-type (a, b) compared to sub-cuticular hyphal growth (white arrowheads) of the Δ*noxA* mutant (c, d) on *L. perenne* leaf sheath and blade. Bars, 50 µm.

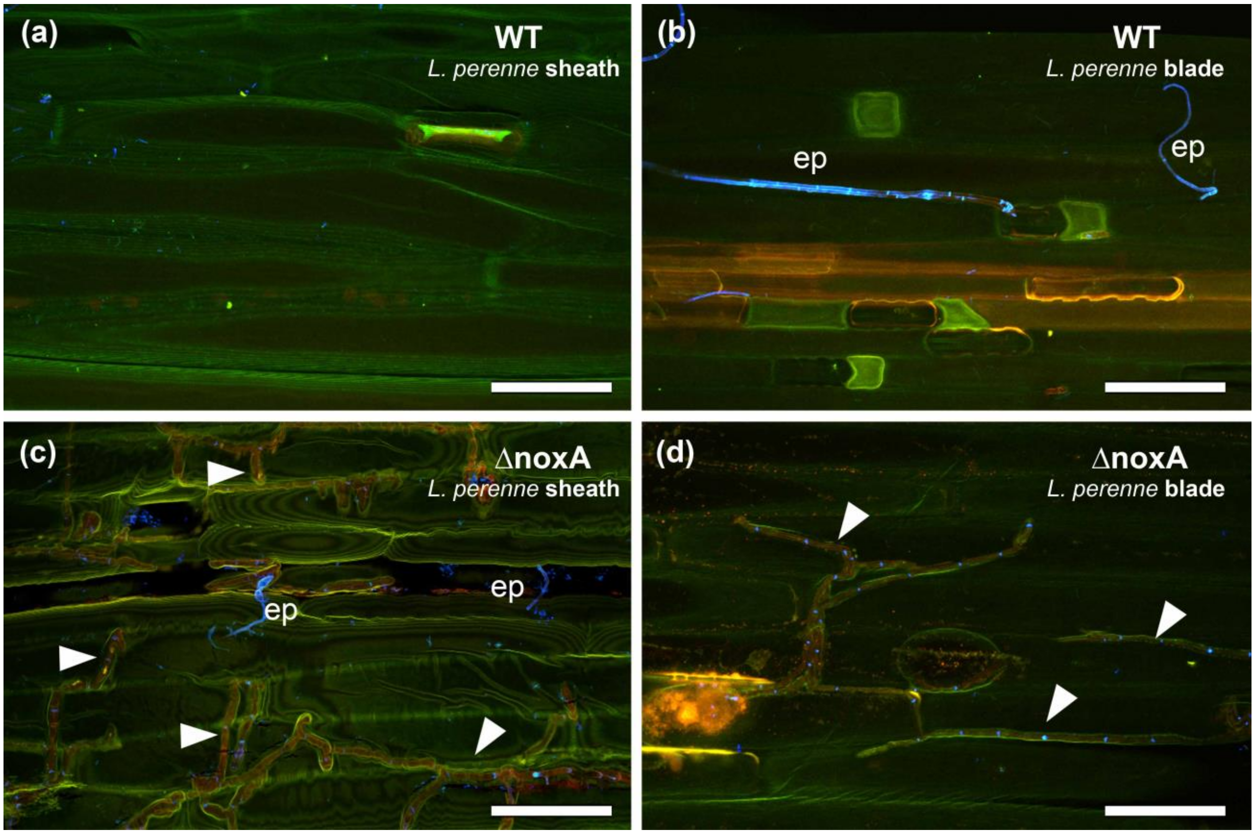

Fig. S9 Epiphyllous growth of NADPH oxidase complex mutants of *E. festucae*. SEM images of hyphae on adaxial blade surface of 8–12-wk-old plants showing epiphyllous hyphae on surface of leaf blade cell division zone. (a) Wild-type infected plant, (b) Δ*noxA* infected plant, (c) Δ*noxB* infected plant, (d) Δ*noxAB* infected plant, (e) Δ*noxR* infected plant. Bars, 50 µm.

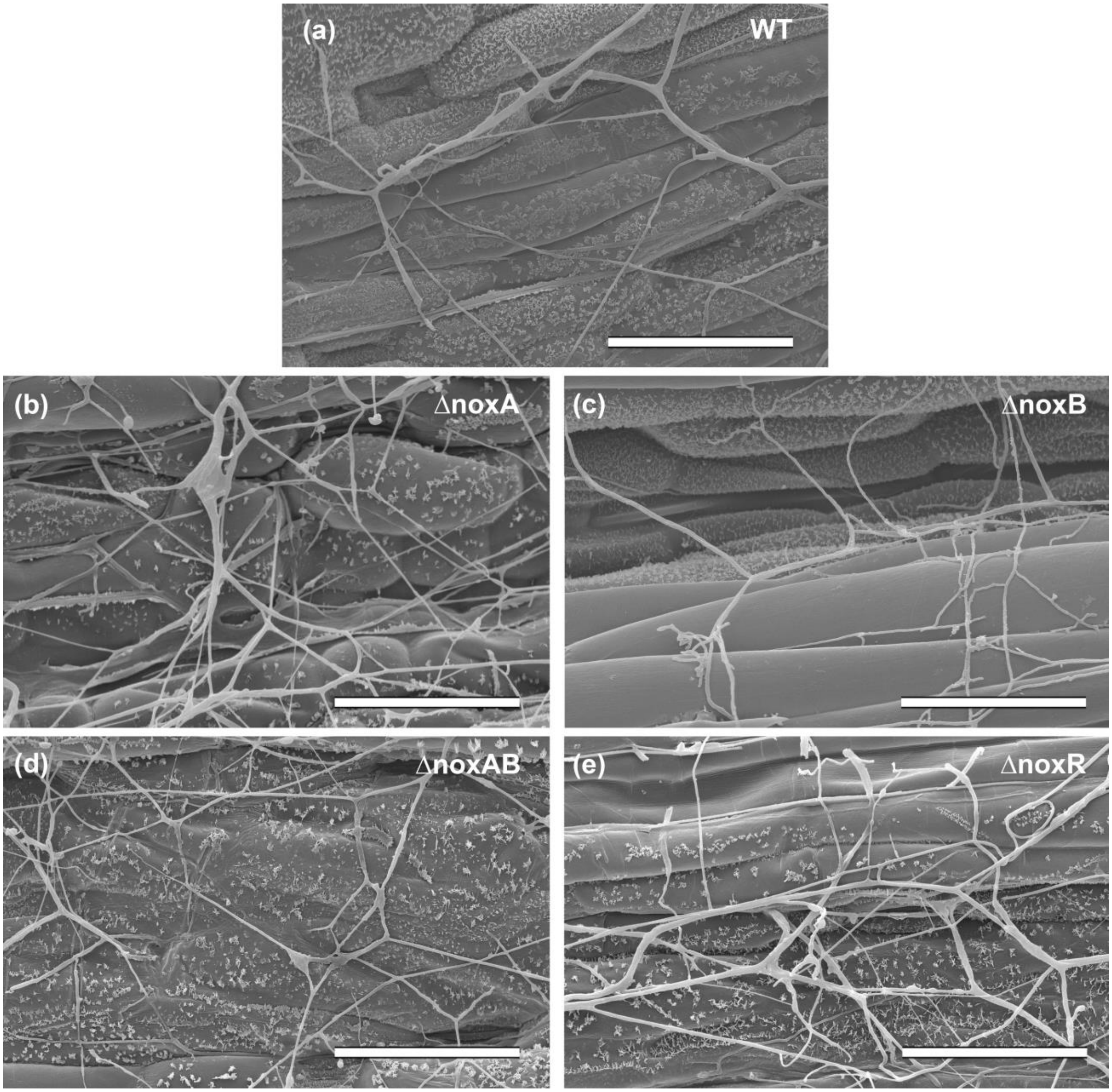

Fig. S10 Sub-cuticular hyphal growth and cuticle breaching of *E. festucae* Δ*noxA* on *L. perenne* leaf blade. (a) Scanning electron microscopy image of hypha on adaxial blade surface. (b) Same hypha as shown in (a) with false coloured plant surface to highlight sub-cuticular growth and cuticle breach. Bars, 1 µm.

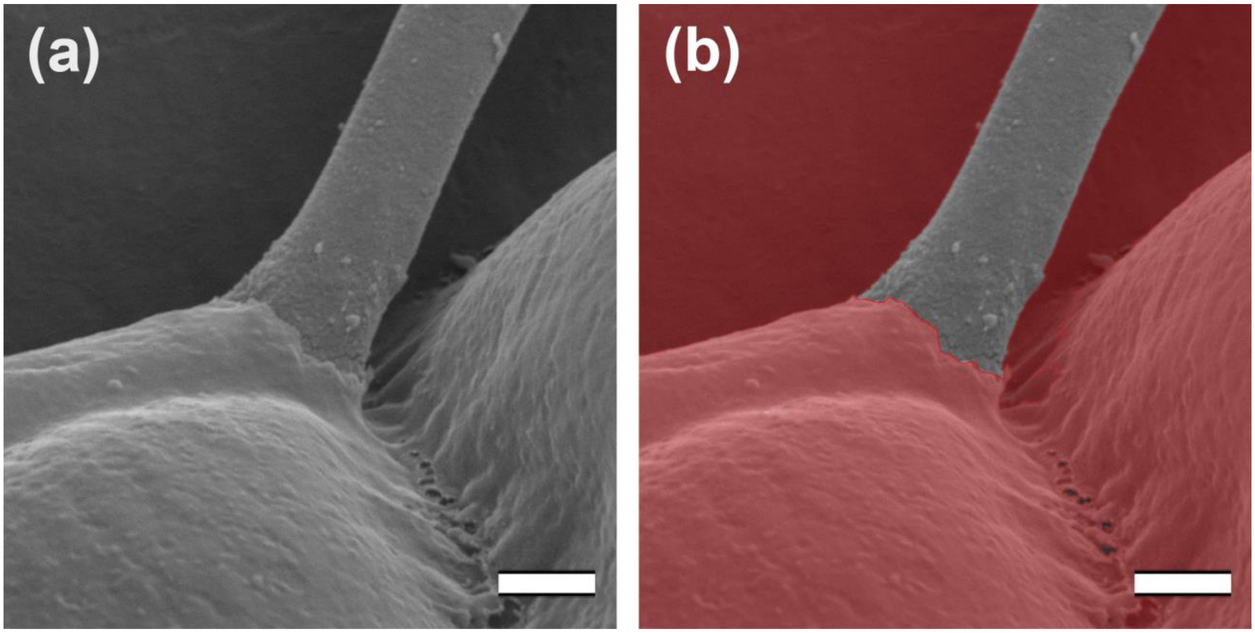

Fig. S11 Wound response of *L. perenne* to presence of *E. festucae* Δ*noxAB* mutant. (a) Hyphae growing between plant epidermis and cuticle after emerging from between epidermal cells (#) and deposition of callose on top of outer periclinal cell wall of infected epidermal cells (black arrow head) and within anticlinal cell walls of adjacent epidermal cells (white arrow head). Two stomata are also depicted. (b) Subset of stacked images of (a) highlighting sub-cuticular hyphae. (c) Similar to (a) and (b) but pseudocolours assigned to WGA-AF488 and aniline blue fluorescence signals have been reversed to highlight presence of chitin (now in red pseudocolour) as detected by WGA-AF488 fluorescence. (d) Sub-cuticular hyphae restricted to a

single plant cell. (e) TEM of Δ*noxA* hypha below plant cuticle showing electron dense area between epidermal cells (black arrowhead), possibly representing plant callose deposition. (a–d) Confocal laser scanning micrographs of aniline blue/WGA-AF488 co-stained blade sample. Bars, 10 µm. (e) Transmission electron micrograph. Bar, 1 µm.

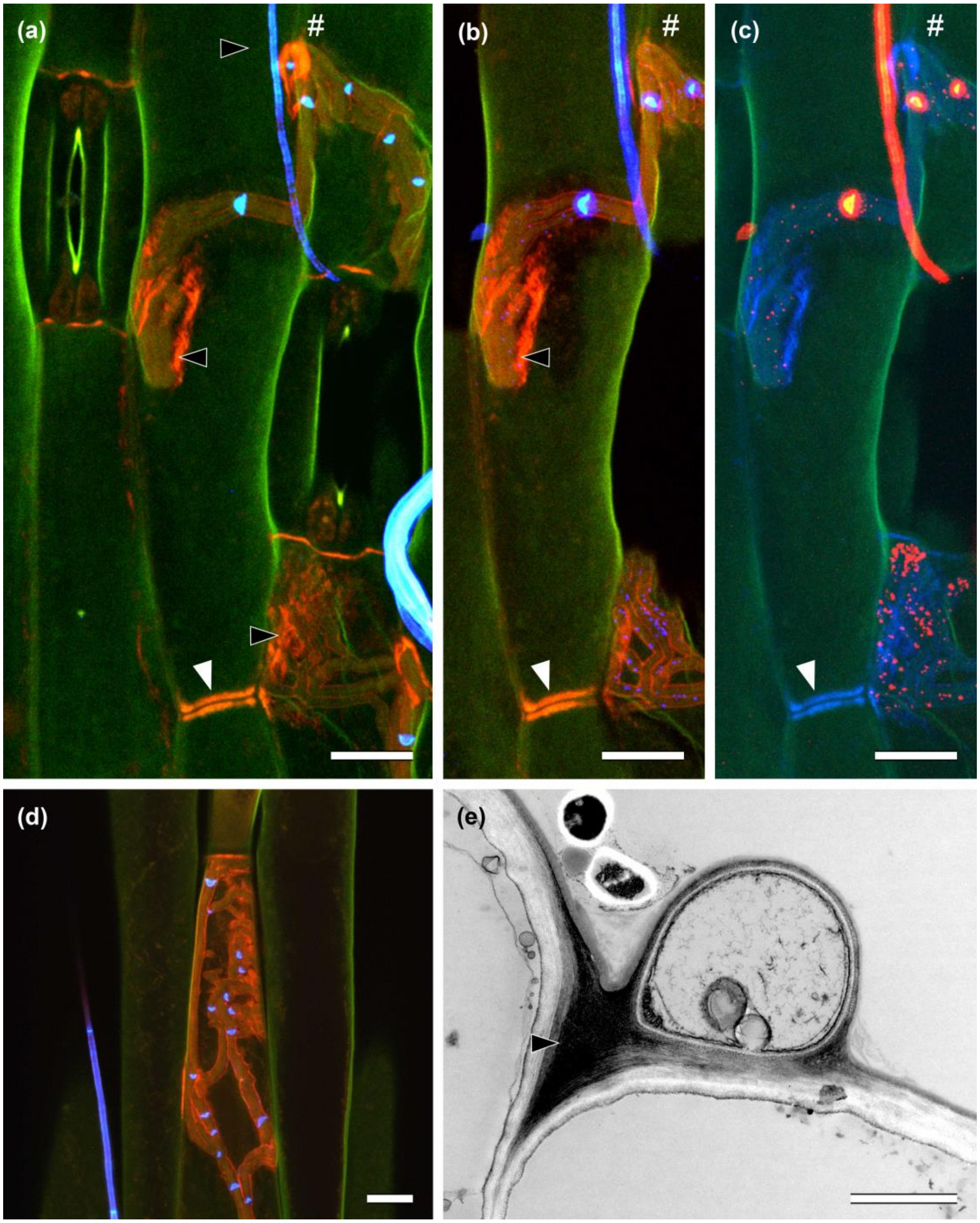

Legends to Movies S1–S4

Movie S1 Movie capturing images taken in the z plane of epiphyllous hyphae and expressorium of wild-type *E. festucae* in symbiosis with *L. perenne* presented in Fig 2(c). Formation of an expressorium on the adaxial leaf blade surface. Z-stack starts from above emerging hypha and epiphyllous hyphal net showing the expressorium penetrating the cuticle and connecting to the endophytic hyphal net. Confocal laser scanning microscopy image of trypan blue stained sample.

Movie S2 Movie capturing images taken in the z plane of an expressorium of wild-type *E. festucae* in symbiosis with *L. perenne* penetrating the host cuticle. Z-stack starts from below cuticle showing polarization of an expressorium at an early stage of host leaf penetration. Confocal laser scanning microscopy image of an aniline blue- WGA-AF488 stained sample.

Movie S3 Movie capturing images taken in the z plane of epiphyllous hyphae and expressorium of wild type *E. festucae* in symbiosis with *L. perenne*. Formation of an expressoria on the adaxial leaf blade surface. Z-stack starts from below emerging hypha and endophytic hyphal net showing the expressorium penetrating the cuticle and epiphyllous hypha. Confocal laser scanning microscopy image of an aniline blue- WGA-AF488 stained sample.

Movie S4 Movie capturing images taken in the z plane of epiphyllous hyphae and expressorium of wild-type *E. festucae* in symbiosis with *L. perenne* presented in Fig 4(b). Formation of an expressorium on the adaxial leaf blade surface. Z-stack starts from below emerging hypha and endophytic hyphal net showing the expressorium penetrating the cuticle and epiphyllous hypha. Confocal laser scanning microscopy image of an aniline blue- WGA-AF488 stained sample.

New Phytologist

Table S1 Biological material

**Plasmids**	**Plasmid design and markers**	**Reference**	**Note**
pPN78 (PN1970)	pBlueScriptII® KS(+) containing *5'noxB*-P*trpCnptII*-T*trpC-3'noxB*; Amp^R /Gen^R	Tanaka *et al.* (2006)	*noxB* replacement vector

**Biological material**	**Relevant characteristics**	**Reference**
**Fungal strains**		
Epichloë festucae		
PN2278 (Fl1)	Wild-type, Isolated from host *Festuca longifolia*	Young *et al*. (2005)
PN2327 (Δ*noxA*)	Fl1/Δ*noxA*::P*trpC-hph*; Hyg^R	Tanaka et al. (2006)
PN3062 (Δ*noxB*#1)	Fl1/Δ*noxB*::*PtrpC-nptII-TtrpC*; Gen^R	This study
PN3063(Δ*noxB*#14)	Fl1/Δ*noxB*::*PtrpC-nptII-TtrpC*; Gen^R	This study
PN3064 (Δ*noxB*#15)	Fl1/Δ*noxB*::*PtrpC-nptII-TtrpC*; Gen^R	This study
PN2469 (Δ*noxB*)	Fl1/Δ*noxB*::*PtrpC-nptII-TtrpC*; Gen^R	Tanaka *et al.* (2006)
PN2470 (Δ*noxAB*)	Δ*noxA*(PN2327)/Δ*noxB::PtrpC-nptII-TtrpC*; Hyg^R; Gen^R	Tanaka *et al.* (2006)
PN2497 (Δ*noxR*)	Fl1/Δ*noxR*::P*trpC-hph*; Hyg^R	Takemoto *et al.* (2006)
**Bacterial strains**		
Escherichia coli		
DH5□	F^-, ϕ80*lacZ*, ΔM15, Δ(*lacZYA-argF*), U169, *recA1*, *endA1*, *hsdR17* (r_k^-, m_k^-), *phoA*, *supE44*, λ^-, *thi-1*, *gyrA96*, *relA1*	Invitrogen

Table S2 Primers used in this study

**Primer**		**Sequence**	**Purpose**
YB151	nox2j	AATGGAGGCAAACGGCGTGA	Δ*noxB* confirmation
YB165	noxB 5r	TCACTGGGAACAACTGGCATG	Δ*noxB* confirmation
YB166	noxB 3r	TGCTCCGTAACACCCAATACGC	Δ*noxB* confirmation
YB167	noxB WT1	GCCACACATGAAGAAAGGATCAT	Δ*noxB* confirmation
YB170	noxB 5f1	TTGTTACCGTCCAGAATCCGA	Δ*noxB* confirmation
YB171	noxB 3r1	CTGCCCTTCTTTCCCATGCT	Δ*noxB* confirmation
YB168	noxB-RF1	AATCAAAGGAAACACGCCGACTAC	*noxB* replacement fragment amplification
YB169	noxB-RF2	AATCAAAGGAAACACGCCGACTAC	*noxB* replacement fragment amplification

References

Takemoto D, Tanaka A, Scott B. 2006. A p67^{Phox}-like regulator is recruited to control hyphal branching in a fungal-grass mutualistic symbiosis. *The Plant Cell* **18**: 2807–2821.

Tanaka A, Christensen MJ, Takemoto D, Park P, Scott B. 2006. Reactive oxygen species play a role in regulating a fungus-perennial ryegrass mutualistic association. *The Plant Cell* **18**: 1052–1066.

Young CA, Bryant MK, Christensen MJ, Tapper BA, Bryan GT, Scott B. 2005. Molecular cloning and genetic analysis of a symbiosis-expressed gene cluster for lolitrem biosynthesis from a mutualistic endophyte of perennial ryegrass. *Molecular Genetics and Genomics* **274**: 13–29.
